# HCV p7 as a novel vaccine-target inducing multifunctional CD4^+^ and CD8^+^ T-cells targeting liver cells expressing the viral antigen

**DOI:** 10.1038/s41598-019-50365-z

**Published:** 2019-10-01

**Authors:** Jonathan Filskov, Peter Andersen, Else Marie Agger, Jens Bukh

**Affiliations:** 1 Copenhagen Hepatitis C Program (CO-HEP), Department of Infectious Diseases, Hvidovre Hospital and Department of immunology and Microbiology, Faculty of Health and Medical Sciences, University of Copenhagen, Copenhagen, Denmark; 2 Department of Infectious Disease Immunology, Statens Serum Institut, Copenhagen, Denmark

**Keywords:** Cellular immunity, Hepatitis C

## Abstract

Despite recent treatment advances for chronic hepatitis C virus (HCV) infection, a vaccine is urgently needed for global control of this important liver pathogen. The lack of robust immunocompetent HCV infection models makes it challenging to identify correlates of protection and test vaccine efficacy. However, vigorous CD4^+^ and CD8^+^ T-cell responses are detected in patients that spontaneously resolve acute infection, whereas dysfunctional T-cell responses are a hallmark of chronic infection. The HCV p7 protein, forming ion-channels essential for viral assembly and release, has not previously been pursued as a vaccine antigen. Herein, we demonstrated that HCV p7 derived from genotype 1a and 1b sequences are highly immunogenic in mice when employed as overlapping peptides formulated as nanoparticles with the cross-priming adjuvant, CAF09. This approach induced multifunctional cytokine producing CD4^+^ and CD8^+^ T-cells targeting regions of p7 that are subject to immune pressure during HCV infection in chimpanzees and humans. Employing a surrogate *in vivo* challenge model of liver cells co-expressing HCV-p7 and GFP, we found that vaccinated mice cleared transgene expressing cells. This study affirms the potential of a T-cell inducing nanoparticle vaccine platform to target the liver and introduces HCV p7 as a potential target for HCV vaccine explorations.

## Introduction

Despite a remarkable success in the development of curative direct acting antiviral treatments for chronic hepatitis C virus (HCV) infection, an effective prophylactic vaccine is considered the only means to substantially reduce the burden of the global HCV epidemic causing at least 400,000 deaths annually^1–3^. Development of a vaccine has, however, proven to be an immense challenge and is hampered by the lack of suitable animal challenge models to test vaccine efficacy, the numerous ways through which the virus evades innate and adaptive immune responses, as well as the extensive genetic diversity of HCV^4,5^. Within an infected individual, HCV exist as a quasispecies often differing by 1–3% at the nucleotide and deduced amino acid level^6,7^. Globally, the genetic heterogeneity, which is around 30% across the HCV major genotypes, further complicates development of an effective pan-genotypic vaccine^8–11^. Potent antibody inducing vaccines have been used against a number of viral diseases, but seem on their own to be insufficient for protection against several viral infections causing chronic diseases, such as HCV, human immunodeficiency virus (HIV) and herpesviruses, which all exhibit a tremendous ability to evade immune surveillance^12–14^.

In addition to vaccines that generate neutralizing antibodies, development of vaccines that also induce specific T-cell responses might be required for viruses that develop into chronic infections, such as HCV^15,16^. Although the main focus in T-cell vaccine development has been on developing strong CD8^+^ T-cell responses, it is now becoming clear that CD4^+^ T-cells comprise an indispensable element of cellular immunity, not only key to establish and maintain multifunctional CD8^+^ T-cells, but also as effectors with direct antiviral potential^17,18^. Further supporting the protective roles of both subsets of T cells, vigorous and broad cellular CD8^+^ and CD4^+^ T-cell responses are observed in individuals who spontaneously clear acute HCV infection^19–23^.

Although the liver acts as a secondary lymphoid organ where priming of T-cells can take place, inefficient stimulatory signals and upregulated inhibitory surface molecules by antigen presenting cells in the liver, skew cellular immunity towards tolerance or apoptosis^24–26^. Ideally, an HCV-vaccine should therefore facilitate efficient peripheral priming of T-cells with the ability to infiltrate and eliminate virus infected hepatocytes^27,28^. At present, the only HCV vaccine being evaluated in clinical trials is a potent CD4^+^ and CD8^+^ T-cell inducing adenoviral vector Chimp/MVA vaccine^29,30^. Alternative vaccine approaches such as peptide- and subunit vaccines are relatively easy to manufacture and store, are considered safe due to their simplicity and can readily be tailored to target- and combine specific antigen sequences^31,32^. These do, however, require co-administration of a suitable adjuvant in order to facilitate cross-presentation of exogenous antigens to MHC class I molecules on antigen presenting cells (APCs), e.g. through Toll-like receptor (TLR) engagement. One such example is the nanoparticle adjuvant, CAF09, that consists of cationic liposomes combined with the immunostimulants monomycolyl glycerol (MMG) and a TLR-3 ligand, Poly(I:C)^33^. This adjuvant efficiently drives antigen specific effector-like CD4^+^ and CD8^+^ T-cell responses with an IFN-γ^+^TNF-α^+^ double positive cytokine profile for a number of different antigens, and has also shown promising results when employed in prophylactic and therapeutic subunit cancer vaccines in preclinical tumor models^33^. The induction of CD8^+^ T-cell responses depend, however, on the route of administration. It appears, that intraperitoneal injection permitting self-drainage from the site of injection to the draining lymph nodes is a prerequisite for the CAF09-adjuvated vaccine particles to reach lymph node resident TLR3^+^ CD8α dendritic cells (DCs) with the capacity to cross present exogenous antigen on MHC class I^34^. Current efforts focus on enhancing the physiochemical characteristics of the adjuvant nanoparticles to target lymph node resident DCs by subcutaneous and intramuscular vaccination routes, which are preferred for human use^35^.

HCV vaccine development has mainly focused on targeting HCV core, envelope- or the nonstructural NS3-NS5B proteins^36,37^. Here, we explore the HCV p7 as a vaccine antigen. This 63 amino acid long polypeptide belongs to the viroporin family that presumably serves as a transmembrane cationic ion channel upon oligomerization^38^. The p7 protein has been found to be crucial for efficient assembly, as well as release of infectious virions, and blocking with specific compounds can abrogate its function^39,40^. Importantly, p7 is also targeted by T cells in HCV patients and immune pressure against p7 can drive mutational escape from T-cell epitopes in chimpanzees experimentally infected with HCV^15,41^. Viral fitness is however tightly linked to alterations in the p7 sequence. Several amino acids of p7 are conserved within HCV genotypes as they are essential for p7 interaction with other genomic regions in a genotype-specific manner^42^. Additionally, regions that are critical for the structural function of the ion channel are highly conserved across genotypes^39^. Thus, p7 might represent an interesting target for antiviral therapy and vaccine development.

Here, we explore the potential of HCV p7 as a vaccine antigen by employing a nanoparticle-based vaccine-approach consisting of a panel of overlapping p7 peptides (pepmix) formulated in the CAF09 adjuvant^43^. The present study demonstrates that p7 is highly immunogenic and can be pursued to induce multifunctional CD4^+^ and CD8^+^ T-cells that efficiently targets the liver and eliminate hepatocytes that express the targeted HCV antigen.

## Results

### HCV p7-specific multifunctional CD4^+^ and CD8^+^ T-cell responses obtained through vaccination in mice

**Figure 1:**
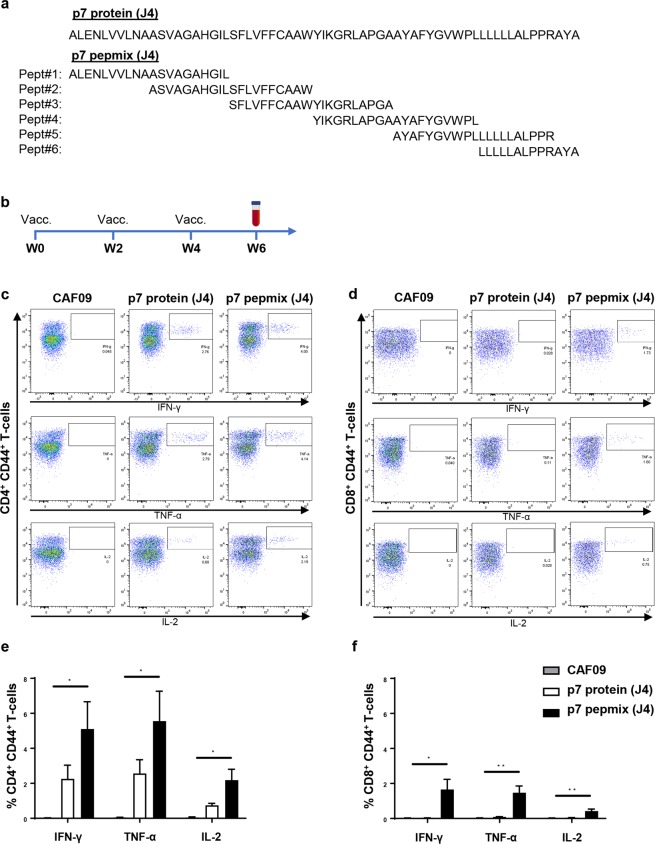
FACS-based frequency analysis of antigen-specific cytokine producing T-cells after vaccination with HCV p7 protein or pepmix. Amino acid sequence of the HCV p7 protein or the six peptides comprising the corresponding pepmix, all based on HCV strain J4 (genotype 1b) (**a**). Vaccine antigens were formulated with the adjuvant CAF09. CB6F1 mice were vaccinated three times at 2-week intervals (**b**); control mice received CAF09 alone. PBMCs isolated from individual mice were re-stimulated with a p7 peptide pool corresponding to the amino acid sequence of the employed vaccine antigen and analyzed by multi-parameter FACS-analysis two weeks after the final vaccination. Representative FACS plots indicate the gating strategy on the IFN-γ (top panel), TNF-α (middle panel) and IL-2 (lower panel) producing population of CD44^+^ CD4^+^ T-cells (**c**) or CD44^+^ CD8^+^ T-cells (**d**). The bar charts show frequencies of p7-specific IFN-γ, TNF-α and IL-2 producing CD44^+^ CD4^+^ T-cells (**e**) and CD44^+^ CD8^+^ T-cells (**f**) out of the total CD4^+^ and CD8^+^ T-cell population, respectively. The data are shown as means and standard errors of the mean (SEM) (n = 4 to 8 mice in each vaccine group). *P < 0.05; **P < 0.01.

To investigate the potential of HCV p7 as a vaccine antigen and its ability to induce CD4^+^ and CD8^+^ T-cell responses, we compared a protein-based approach with an approach based on six overlapping peptides (pepmix) covering the entire p7 sequence of HCV strain J4 (genotype 1b)^44^. Figure 1a shows the amino acid sequence of the p7 protein (J4) and peptides contained in p7 pepmix (J4). The vaccine antigens were formulated in the cationic adjuvant CAF09^33^. CB6F1 mice were immunized intraperitoneally three times at two-week intervals and the immune response was assessed two weeks after the final vaccination, as indicated on the timeline shown in Fig. 1b.

To assess the p7-specific T-cell responses, splenocytes were isolated and re-stimulated with a pool of the six overlapping p7 peptides used in the pepmix vaccination, followed by intracellular (IC) flow cytometry-analysis. Representative plots of CD44^+^CD4^+^ (Fig. 1c) and CD44^+^CD8^+^ (Fig. 1d) T-cells producing IFN-γ (upper panel), TNF-α (middle panel) and IL-2 (lower panels) are shown for controls (CAF09), as well as p7 protein (J4) and p7 pepmix (J4) vaccinated mice. Whereas the p7 protein (J4)-based vaccine approach only induced antigen-specific CD4^+^ T-cells producing IFN-γ, TNF-α and IL-2, the p7 pepmix (J4) vaccine generated both robust CD4^+^ and CD8^+^ T-cell responses (Fig. 1e,f). Notably, escalating the p7 protein dose to equimolar antigen levels of peptides used in the p7 pepmix vaccine did not lead to induction of CD8^+^ T-cells (Supplementary Fig. S1).

### p7 protein and p7 pepmix (J4) induced a similar repertoire of epitope specific CD4^+^ T cells

**Figure 2:**
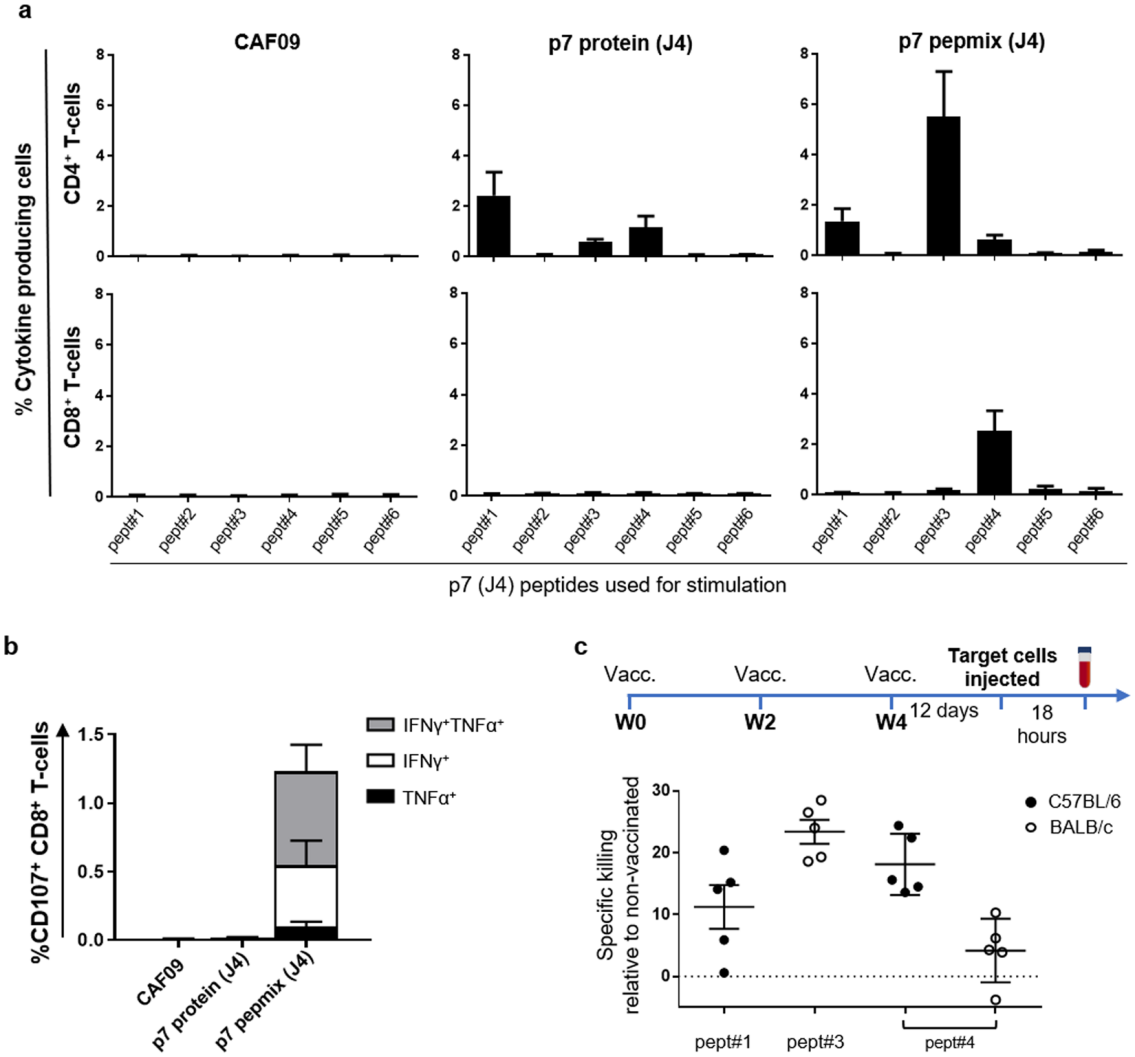
Specific killing by cytotoxic CD4^+^ and CD8^+^ T-cells *in vivo* (**a**) Mice were vaccinated three times at 2-week intervals with HCV p7 protein or pepmix (strain J4) as indicated above the graphs. Splenocytes from individual mice were isolated two weeks after the final vaccination and re-stimulated with each of the individual peptides spanning the p7 (J4) sequence to map the repertoire of epitope-specific responses. The peptide# identifiers are indicated below the *x-*axis. Bars indicate the total frequency of epitope-specific CD44^+^ CD4^+^ T-cells (upper panels) and CD44^+^ CD8^+^ T-cells (lower panels) able to produce IFN-γ, TNF-α or IL-2 in any combination. Data are shown as means and SEM (n = 4 to 6 mice in each vaccine group). (**b**) Pept#4 (J4)-specific CD8^+^ T-cells were further assessed for their ability to co-express CD107 with IFN-γ and/or TNF-α. (**c**) CFSE-labelled target cells from naïve mice were pulsed with single p7 peptides #1, #3 or #4 or left untouched and subsequently injected i.v. into controls and p7 pepmix (J4) vaccinated C57BL/6 or BALB/c mice 12 days after the final immunization, as indicated on the timeline. Eighteen hours after i.v. injection, the remaining population of target cells in in controls or vaccinated mice were identified based on their CFSE-fluorescence and MHC-II expression (timeline). The graph shows percent specific killing of target cells pulsed with peptides #1, #3 or #4 in individual mice vaccinated with p7 pepmix relative to control mice. Means and SEM are indicated on the graph (n = 5 mice in each vaccine group).

We have previously reported that vaccination with peptide panels could induce a broadened T cell response in a given genetic context^43^. To determine differences in the repertoire of epitope-specific T-cells induced with p7 protein (J4) compared to pepmix (J4) in a CB6F1 background, splenocytes from vaccinated mice were stimulated with each of the individual J4 peptides as indicated below the graphs in Fig. 2a. Here, we found that both vaccines generated CD4^+^ T-cell responses targeting epitopes within peptide #1, #3 and #4 (Fig. 2a, upper panel), whereas CD8^+^ T-cells recognizing peptide #4 were detected only after p7 pepmix (J4) vaccination (lower panel). An experiment in which mice were vaccinated with p7 pepmix based on HCV strain H77 (genotype 1a), revealed CD4^+^ and CD8^+^ T cells only targeting H77 peptide #4 (Supplementary Fig. S2). *In silico* prediction indicated the presence of strong CD8^+^ T cell epitopes within both the J4 and H77 sequence of the corresponding #4 peptide and that these were linked to a C57BL/6 background (Supplementary Table S1). The majority of peptide #4 specific CD107^+^ CD8^+^ T-cells were multifunctional in their capacity to co-produce IFN-γ and TNF-α indicating enhanced cytotoxic potential (Fig. 2b)^45^.

### HCV p7 pepmix (J4) induced T-cells with *in vivo* cytotoxic capability

Next, we used the information of epitope-specific cellular responses to further evaluate the functional capabilities of CD4^+^ and CD8^+^ T-cells by assessing their cytotoxic capacity *in vivo*. To further decipher the MHC-restriction of targeted epitopes, we here immunized C57BL/6 or BALB/c mice with p7 pepmix (J4) rather than employing the CB6F1 offspring cross. CD4^+^ T cell epitopes within peptide #4 were recognized in both mouse strains, whereas the epitope within peptides #1 and #3 was restricted to C57BL/6 and BALB/c backgrounds, respectively (Supplementary Figs S3a,b versus S4a,b). CD8^+^ T cell responses were only observed in C57BL/6 mice targeting peptide #4.

Knowing which peptides contain epitopes targeted by vaccination, we pulsed CFSE-labelled target cells with peptide #1, #3 or #4 and subsequently injected them i.v. into p7 pepmix (J4) vaccinated C57BL/6 or BALB/c mice as indicated on the timeline in Fig. 2c. After eighteen hours, splenocytes were retrieved and vaccine-induced killing by T-cells specific for peptide #1, #3 or #4 was determined by measuring the frequencies of remaining target cells in vaccinated versus control mice by flow cytometry-analysis, and gating on CFSE-labelled cells (Supplementary Figs S3c and S4c). C57BL/6 mice eliminated 18.1% ± 2.2 of target cells pulsed with peptide #4 compared to 4.2% ± 2.3 in BALB/c mice (Fig. 2c). This corresponds well with the strong CD8^+^ and CD4^+^ T cell immunity observed in C57BL/6 mice and absent CD8^+^ T cells in BALB/c mice. Nevertheless, elimination of target cells pulsed with peptide #1 (11.2% ± 3.5) or #3 (23.4% ± 2.0), which only contain CD4^+^ T-cell epitopes, suggests that effector mechanisms other than CD8^+^ T-cells contribute to elimination of target cells *in vivo*.

### Vaccine-induced clearance of liver cells co-expressing HCV-p7 and GFP

In addition to inducing vigorous, multifunctional cellular immunity with cytotoxic capabilities, an effective HCV-vaccine should also induce T-cells with the ability to infiltrate the liver environment in order to eliminate viral infected hepatocytes. This is, however, a challenge not possible to address in any immune competent animal model other than chimpanzees^5,46^. Generation of transiently liver-transgenic mice by hydrodynamic injection of DNA solultion has been used as a valuable tool to assess vaccine efficacy in terms of T-cell migration to the liver and elimination of hepatocytes expressing HCV NS3/4A^27,47–50^. To ensure liver specific expression of target proteins, we modified this approach by constructing plasmids encoding HCV-p7 (J4 or H77) and GFP expressed from a single albumin promoter and its upstream enhancer sequence^51^. Further, to obtain stochiometric expression of the transient transgenes, the p7 and GFP sequences were separated by the thosea asigna T2A linker sequence, a foot and mouth disease virus (FMDV) 2A-like self-cleaving protease^52,53^. Initially, mice received PBS, or 1, 10 or 50 µg p7(J4)-GFP plasmid by hydrodynamic injection and we subsequently assessed the liver for GFP-expressing cells by flow cytometry-analysis three days after the injection. In mice that received the highest dose (50 µg DNA), we found 1.8% ( ± 0.7) GFP^+^ cells. Importantly, in these mice, no GFP expression was observed in the kidney, heart, lung or spleen (Supplementary Fig. S5a). Next, after hydrodynamic injection with PBS (Supplementary Fig. S5b, upper panel) or 100 µg p7(J4)-GFP (lower panel) we visualized GFP expressing liver cells by fluorescence microscopy after co-staining with DAPI. In parallel, unstained cells from these mice were run on flow cytometry to identify GFP-expressing cells based on their fluorescence (Supplementary Fig. S5c). Comparable results could be obtained by hydrodynamic injection of a similar plasmid construct expressing p7 based on HCV strain H77 (Supplementary Fig. S5d).

**Figure 3:**
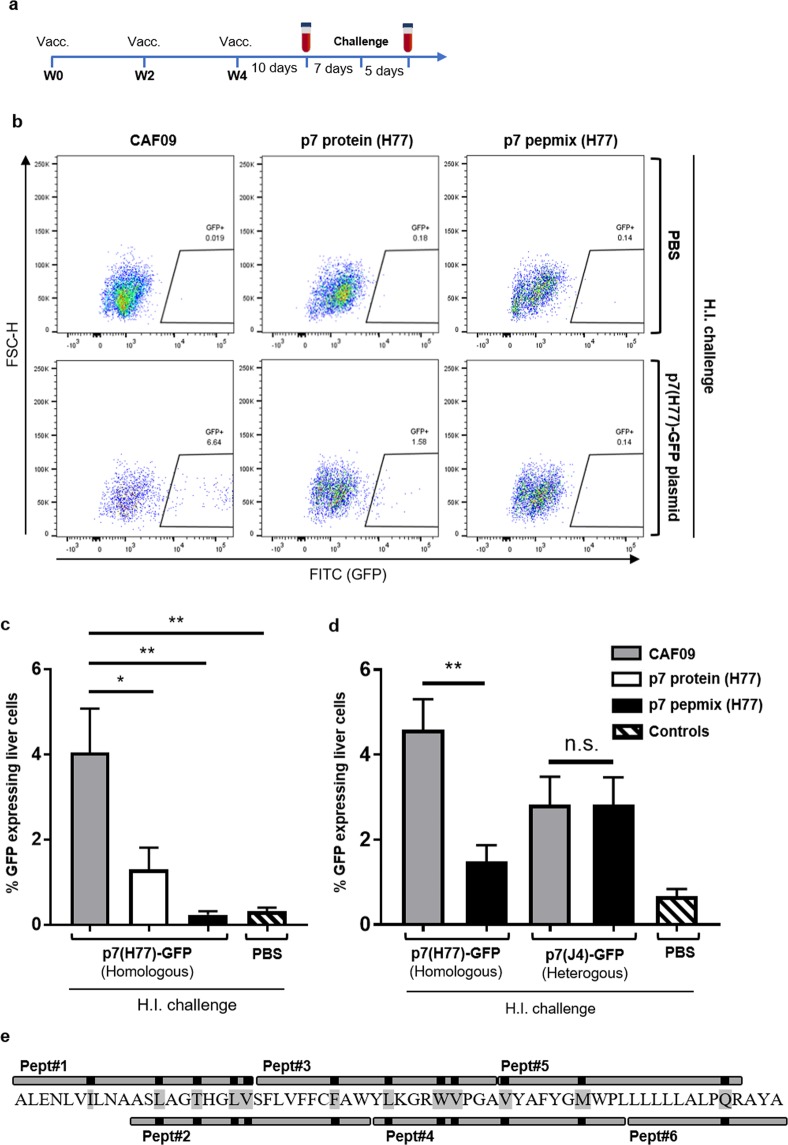
HCV p7 vaccination reduced GFP-expression in the liver after surrogate challenge. (**a**) The diagram indicates time points for vaccination, bleeding, surrogate challenge and termination of the experiment. (**b**) Representative FACS-plots shows gating of GFP-expressing liver cells from CAF09 versus p7 protein and pepmix (strain H77) vaccinated mice five days after hydrodynamic injection (H.I.) of PBS (upper panel) or 100 µg p7(H77)-GFP plasmid (lower panel). (**c**) The bar charts show frequencies of GFP-expressing liver-cells in vaccinated mice after surrogate challenge. Control mice that received hydrodynamic injection with PBS alone, consisted of 3-4 mice from each vaccine group. Bars represent means and standard errors of the mean (SEM) (n = 7 to 12 mice in each group). *P < 0.05; **P < 0.01. (**d**) Reactivity of p7 pepmix (H77)-induced immunity with liver cells challenged with either p7(H77)-GFP or p7(J4)-GFP plasmid (homologous vs. heterologous relative to the vaccine antigen) as indicated below the chart, was performed 4 days after hydrodynamic injection. Naïve control mice were challenged with PBS alone. Bars represent means and standard errors of the mean (SEM) (n = 7 to 8 mice in each vaccine group; 3 naïve controls received PBS by H.I.). **P < 0.01. (**e**) Schematic presentation of the HCV p7 pepmix vaccine with the six overlapping peptides aligned with the p7 (H77) amino acid sequence. Black marks and amino acids shaded with grey indicate sequence variations compared to the J4 strain.

We next evaluated vaccine efficacy by investigating the ability of HCV p7 specific T-cells in clearing liver cells. Mice were vaccinated with p7 protein (H77) or p7 pepmix (H77) as outlined in Fig. 3a. Seventeen days after the final vaccination, mice were challenged with 100 μg p7(H77)-GFP plasmid DNA by hydrodynamic injection. Controls received PBS only. After another five days, flow cytometry-analysis was used to assess the ability of vaccinated mice in clearing liver cells co-expressing p7 and GFP. Representative plots show the frequency of GFP-expressing liver cells in vaccinated mice that received hydrodynamic injections with PBS (Fig. 3b, upper panel) or were challenged with p7(H77)-GFP plasmid (Fig. 3b, lower panel). CAF09 immunized control mice injected with either PBS or plasmid were used to outline gates defining GFP^-^ vs. GFP^+^ liver cells based on their fluorescence intensity. We found that p7 pepmix (H77) vaccinated mice cleared transiently transfected GFP^+^ liver cells to a level comparable to the background signal in control mice that received PBS by hydrodynamic injection (Fig. 3c). Further, p7 protein (H77) vaccination resulted in a lower but still significant reduction of GFP^+^ cells (Fig. 3c).

As seen in CAF09 immunized mice, transiently transfected liver cells within the GFP^+^ gate differed in their level of GFP expression (Fig. 3b, lower panel, left). Interestingly, it was evident that p7 protein vaccinated mice efficiently targeted cells that expressed high levels of transgenes, whereas liver cells within the GFP^+^ gate, but with a lower expression of GFP were cleared to a lesser extend (Fig. 3b, lower panel, middle).

Additionally, to assess the potential of p7 pepmix in cross-protecting against a heterogenous genotype, we vaccinated mice with p7 pepmix (H77) and subsequently performed hydrodymic challenge with either p7(H77)-GFP or p7(J4)-GFP. Here, we found that significant reduction of GFP^+^ cells could be achieved four days after challenge with the homologous p7(H77), but not the heterologous p7(J4) sequence (Fig. 3d). The lack of cross-reactivity in vaccinated mice followed by heterologous challenge may be ascribed to differences between the H77 and J4 p7 amino acid sequences, which is apparent within each of the six p7 pepmix peptides (Fig. 3e).

### HCV p7-specific T cell responses induced by vaccination pre- and post- antigen challenge

**Figure 4:**
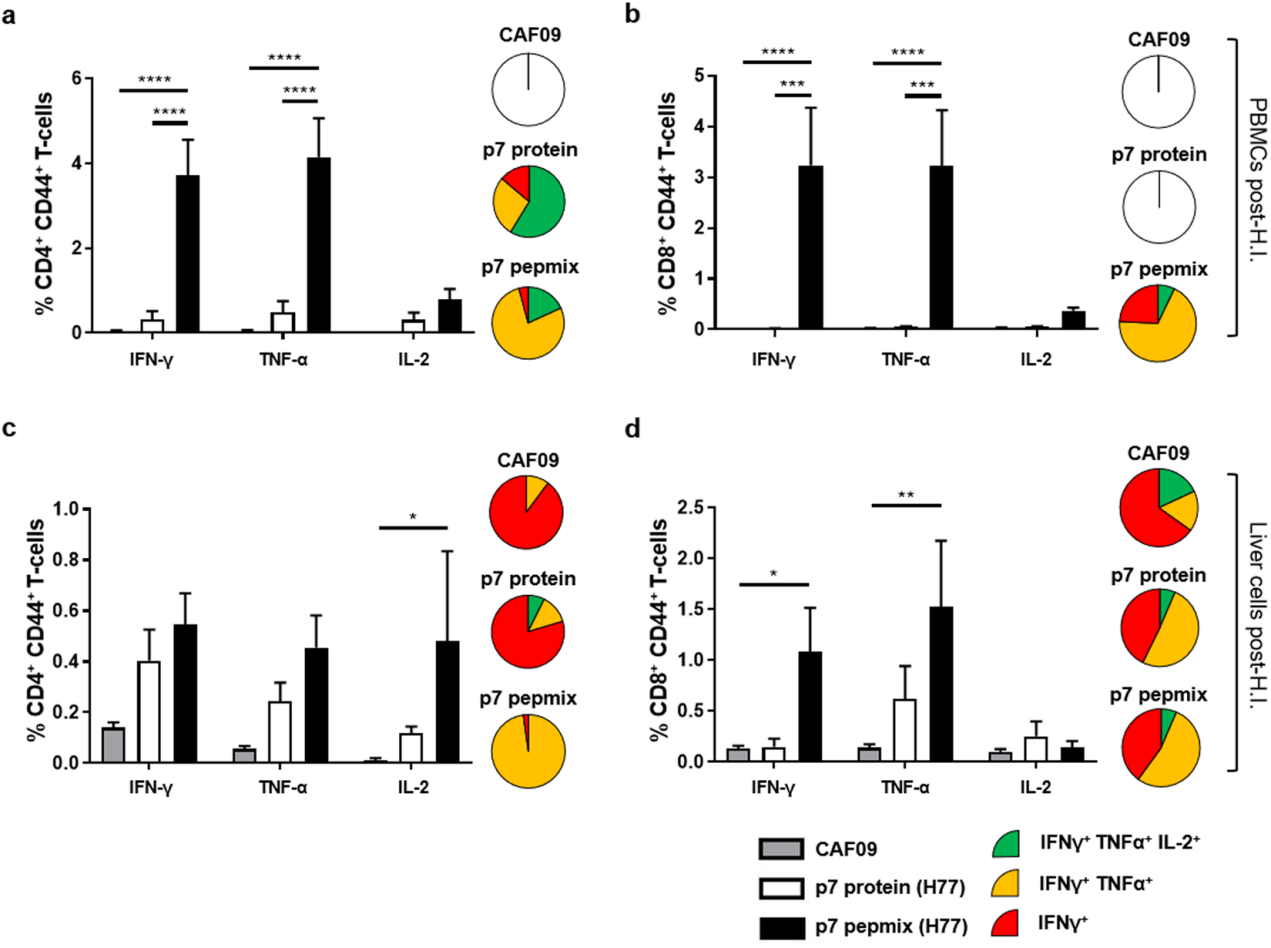
Frequencies and functional characterization of antigen-specific cytokine producing T-cells from HCV p7 protein or p7 pepmix (H77) vaccinated mice after surrogate challenge. Five days after challenge with p7(H77)-GFP plasmid, PBMCs (**a**,**b**) and liver cells (**c**,**d**) were isolated from individual mice, re-stimulated with a pool of overlapping p7(H77) peptides, and analyzed by multi-parameter IC-FACS to assess the frequencies of p7-specific IFN-γ, TNF-α and IL-2 producing CD44^+^ CD4^+^ T-cells (**a**,**c**) or CD44^+^ CD8^+^ T-cells (**b**,**d**) out of the total CD4^+^ or CD8^+^ T-cell population, respectively, 5 days after surrogate challenge. Pie charts show the distribution of IFN-γ producing CD44^+^ CD4^+^ or CD44^+^ CD8^+^ T-cells divided into three distinct subpopulations based on their ability to co-produce TNF-α alone or in combination with IL-2. Empty pies indicate total cytokine responses below a 0.2% cut-off. Data are shown as means and standard errors of the mean (SEM) (n = 7 to 12 mice in each vaccine group). *P < 0.05; **P < 0.01; ***P < 0.001; ****P < 0.0001.

Given the efficient clearance of antigen presenting target cells in p7 pepmix (H77) vs. p7 protein (H77) vaccinated mice, we also evaluated the induced T cell responses after challenge. We detected robust CD4^+^ and CD8^+^ T-cell responses after p7 pepmix (H77) vaccination, wheras a considerably lower CD4^+^ T cell response was seen in p7 protein (H77) vaccinated mice (Fig. 4a,b, bars).

Compared to pre-challenge responses, we observed elevated magnitudes of cytokine producing CD4^+^ T-cells in p7 pepmix vaccinated mice, whereas CD8^+^ responses remained unaltered (compare to Supplementary Fig. S6a,b). It is unclear to which extent the magnitude of T cell responses are influenced by the challenge or merely reflects the kinetics of the vaccine induced response.

Boolean gating revealed that the majority of antigen specific CD4^+^ and CD8^+^ T-cells in p7 pepmix vaccinated mice were able to co-produce INF-γ and TNF-α (Fig. 4a,b, pies), indicating that the response is skewed towards an effector-like response, whereas a larger proportion of IFN-γ, TNF-α and IL-2 co-producing CD4^+^ T-cells observed in p7 protein vaccinated mice suggest a response with proliferative potential.

Detailed analysis on CD4^+^ T-cells in mouse models propose the ability of proliferative PD-1^hi^KLRG1^lo^ to differentiate into high-capacity cytokine producing PD-1^int^KLRG1^hi^ cells^54^. We therefore analyzed the PD-1 and KLRG1 expression on cytokine producing T-cells and found CD4^+^ T cells in p7 pepmix (H77) vaccinated mice to be skewed towards a PD-1^int^KLRG1^hi^ phenotype after surrogate challenge (Supplementary Figs S6c vs. S7a). Compared to their PD-1^hi^KLRG1^lo^ counterparts, these cells were also far more efficient in producing INF-γ and TNF-α (Supplementary Figs S6e and S7c). Contrary, the PD-1^hi^KLRG1^lo^ subset, that clearly was the dominating one in p7 protein (H77) vaccinated mice, expressed higher levels of IL-2 and CD62L. Such pattern of differential cytokine production linked to PD-1 and KLRG1 expression was not as apparent on the CD8^+^ T-cell level (Supplementary Figs S6d,f and S7b,d).

Compared to PMBCs, the frequncy of antigen-specific CD4^+^ T-cells found in the liver was considerably reduced in p7 pepmix (H77) vaccinated mice (Fig. 4c). Here, cytokine producing cells displayed a lower degree of multifunctionality than circulating T-cells (pie charts). Interestingly, minute CD8^+^ T-cell responses were detected in the liver of p7 protein (H77) vaccinated mice after challenge (Fig. 4d). Contrary, no p7 specific CD8^+^ T-cells were detected in this vaccine group when PBS only was administered by hydrodynamic injection (Supplementary Fig. S8). Taken together, our data suggest that p7 protein and pepmix induce T-cell responses that differ not only in magnitude but also display distinct cytokine profiles and functionalities.

## Discussion

In the present study, we have established HCV p7 as a potential vaccine antigen. Our data show that multifunctional CD4^+^ and CD8^+^ T-cell responses could be generated by vaccination with overlapping peptide panels covering HCV p7 sequences formulated in cross-priming, cationic nanoparticles. Further, using a developed surrogate challenge model we demonstrated that vaccinated mice could clear liver cells expressing HCV p7.

Despite the heterogeneity of p7 across HCV genotypes, certain amino acids within the transmembrane helices and cytoplasmic loop are highly conserved across HCV genotypes^39,42^. Cyclic amino acids such as tryptophan (W) and tyrosine (Y) are considered important structure-function determinants for viroporins of influenza A and HIV^55,56^. This may also be the case for HCV p7 as substitution of the highly conserved tryptophan and tyrosine residues at position 776 and 788, respectively, dramatically reduced infectivity and release of new virions^39^. Several other residues of p7 are subject to mutational escape from T-cells as demonstrated in chimpanzees experimentally infected with HCV^15,41,57^. Although confounding factors that potentially mask detection of p7 specific T-cells in peptide re-stimulation assays include the presence of non-responsive, exhausted T cells or re-stimulation with non-autologous viral sequences, immune pressure by T-cells targeting p7 has also been demonstrated in patients chronically infected with HCV^58^. Here the epitope sequence FYGMWPLL (residue 790–797 of H77) is recognized in patients with a Cw7 or A29 HLA background. Interestingly, several studies of chimpanzees infected with different HCV clones showed that immune selection pressure led to substitution of the methionine (M) residue found within this region^15,41,57^. These observations could indicate the presence of one or more promiscuous T-cell epitopes recognized across a broad genetic background. In this context, it is also noticeable that the sequence FYGMWPL (H77) and FYGVWPL (J4) as well as the conserved tyrosine residue 788 is present in peptide #4 of the HCV p7 pepmix, which induced both CD4^+^ and CD8^+^ T cell responses in CB6F1 mice (Fig. 2 and Supplementary Fig. S2). Thus, although broad T-cell responses cannot be induced with HCV p7 due to its relatively small size, it represents an interesting vaccine antigen that should be examined further and might be useful in combination with other antigens.

Vaccine strategies pursuing to target additional epitopes not targeted by an infection-driven response have been used in several systems against HCV, HIV, lymphocytic choriomeningitis virus (LCMV), human papillomavirus (HPV) and *M*. *tuberculosis* (*M*.*tb*.). Such strategies exploit truncated or fragmented antigens rather than injection- or expression of full-length proteins to influence antigen processing and presentation by APCs^43,59–64^. Further, substitution or removal of certain amino acid residues or sequences flanking potential T-cell epitopes have been shown to enhance induction of particularly CD8^+^ T-cell responses, which would otherwise not be supported through natural processing of the corresponding full-length antigen^65–67^. Concordant with these observations, immunization with the full-length HCV p7 protein only induced CD4^+^ T-cells, whereas p7 pepmix consisting of 6 overlapping peptides of the identical sequence generated robust CD4^+^ and CD8^+^ T-cell responses.

Whereas multifunctional, cytotoxic CD8^+^ T-cells are regarded as the primary mediators of clearance of HCV infected cells, the role of cytotoxic CD4^+^ T-cells is less well studied although there is evidence of immune pressure on the HCV genome exerted by these cells^17^. It has, however, become clear that specialized CD4^+^ T-cell effector subsets with direct cytolytic capacity can be induced to target a number of viral infections, e.g. HIV, poliovirus and influenza in a granzyme B, perforin or FasL mediated manner^68–71^. Accumulation of cytotoxic CD4^+^ T-cells is also seen in persistent HCV infection^72^. Exposure to high levels of antigen in the presence of IL-2 appears to favor differentiation into cytotoxic CD4^+^ T-cells, which typically co-express IFN-γ and TNF-α^73^. In our study, vaccination with HCV p7 pepmix generated CD4^+^ T-cells with a distinguished IFN-γ^+^TNF-α^+^ double positive cytokine profile and a phenotypic effector-like signature.

We here found that individual transfected liver cells within the GFP^+^ gate differed in their level of GFP expression and thus presumably also expression of the HCV p7 protein (Fig. 3b). Although the HCV p7 protein vaccinated mice could target p7 and GFP co-expressing cells, it was evident, that GFP^+^ cells expressing higher levels of transgenes, were more efficiently cleared than GFP^+^ cells with a lower expression of transgenes. It is therefore likely that induction of CD8^+^ T-cells is a prerequisite for elimination of liver cells co-expressing lower levels of p7 and GFP, which could explain the enhanced clearance of GFP^+^ liver cells in p7 pepmix vaccinated mice.

Although vaccination with HCV p7 protein only induced CD4^+^ T-cell responses, minute CD8^+^ T-cell responses were detected in the liver of these mice, but only after hydrodynamic challenge with plasmid-DNA (and not PBS). As IL-2 producing CD4^+^ T-cells play a central role in antiviral immunity, also by providing help to induce and maintain CD8^+^ T-cell immunity, it could therefore be speculated whether CD4^+^ T-cells induced through protein vaccination was a prerequisite for induction of CD8^+^ T-cells upon endogenous p7-expression^74^.

CD8^+^ T-cells may therefore account for clearance of HCV p7-GFP expressing liver cells in both p7 protein and pepmix vaccinated mice. Direct interaction of CD4^+^ T-cells with target cells requires expression of MHC class II which is not normally expressed on parenchymal cells. However, aberrant expression of MHC class II by hepatocytes has been observed during clinical hepatitis^75,76^, hepatocellular carcinoma (HCC)^77,78^, in HCC cell lines^79,80^ or primary hepatocytes under inflammatory stress conditions^81^. Endogenous expressed antigens can readily enter the MHC class II pathway, and hepatocytes expressing MHC class II together with co-stimulatory B7.1 molecules (CD80) have been shown to activate both Th1 and Th2 CD4^+^ T-cells in a mouse model^82,83^. Thus, although the prerequisites for direct hepatocyte-CD4^+^ T-cell interaction may be met, it remains to be determined whether hepatocytes infected with HCV can present viral antigens on MHC class II and hereby be targeted directly by CD4^+^ T-cells.

As the CAF09 adjuvant also supports induction of humoral responses, other effector mechanisms such as antibody-dependent cellular cytotoxicity (ADCC), may also contribute to the clearance of target cells *in vivo*^84,85^. Although the contribution of ADCC in clearance of HCV remains to be studied in detail, ADCC represents an important asset in cancer immunotherapy and is also believed to contribute to clearance of HIV infection^86,87^.

It would be of interest in future studies to address these questions experimentally, e.g. in a fully immune competent small animal challenge model with an HCV-related hepatotropic virus, such as the Norway rat hepaci virus (NrHV)^88,89^. Further, this model could serve as an important tool for future evaluation of a NrHV-based p7 pepmix vaccine, e.g. also combined with other viral antigens, and its ability to overcome viral immune evasion mechanisms including mutational escape. The present study serves as a proof of principle that efficient cellular immunity against HCV p7 can be induced by vaccination with peptide-based cross-priming nanoparticles. Importantly, by including additional peptides in the library, the pepmix vaccine can be expanded to cover multiple HCV sub- or genotype sequences of p7. Finally, this approach could be further developed by targeing p7 in combination with other antigens, such as peptides covering HCV NS3, to ensure generation of a broader T-cell repertoire targeting several conserved regions of the virus, which is anticipated to be a prerequisite for efficient protection against HCV^43^.

## Materials and Methods

### Mice

Six to eight week old female CB6F1 (C57BL/6 X BALB/c), C57BL/6, and BALB/c mice were purchased from Harlan Scandinavia, and housed in the experimental-animal facilities at Statens Serum Institut. All handling and procedures were performed by authorized personnel in accordance with the European community Directive 86/609 and approved by the Danish Council for Animal Experiments.

### Vaccines

p7 deduced from the cloned HCV strains J4 (genotype 1b)^44,90^ or H77 (genotype 1a)^91^ were used as vaccine antigens. Panels of five 20-mer and one 13-mer peptides covering the entire p7 sequence of J4 or H77 were used in the pepmix vaccines and the corresponding full-length p7 proteins were synthesized as polypeptides. Lyophilized peptides were reconstituted in DMSO (10 mg/ml). Vaccine antigens were prepared with the cationic adjuvant formulation CAF09 (250 µg DDA/50 µg MMG/50 µg Poly(I:C)/dose). Unless stated otherwise, mice received 10 µg p7 protein or 10 µg of each peptide in pepmix vaccines. All vaccines were administrated intraperitoneally at a volume of 0.2 ml, three times at two-week intervals.

### Cell preparations

Blood was obtained by periorbital puncture or via the facial vein, and PBMCs were isolated by gradient centrifugation with Lympholyte (Cedarlane) as per manufacturers protocol. Splenocytes and cells from kidneys were obtained by homogenization through a 100 µm nylon cell strainer followed by hypotonic lysis of red blood cells in 0.84% NH_4_Cl and two washes with PBS. Cells from lung and heart were isolated by the same procedure as described above, following digestion of tissue in 1 mg/ml collagenase IV for 45 minutes. Livers were perfused and homogenized for 2 × 25 seconds on gentle MACS dissociator in C-tubes containing 5 ml RPMI, 0.4 mg/ml collagenase IV and 0.5 µg/ml DNase 1, and the tissue suspensions was then incubated 25 minutes at 37 °C with frequent shaking. The suspension was then forced through a 100 µm cell strainer, washed in PBS, and resuspended in 4.5 ml 0.84% NH_4_Cl. After five minutes, liver cells were washed in twice in RPMI. Cells from any tissue were resuspended in media (RPMI 1640 supplemented with 1 mM glutamine, 1% pyruvate, 1% penicillin-streptomycin, 1% Hepes and 10% FBS) and cell cultures were performed in 96-well V-bottom plates.

### Plasmids

VectorBuilder 1.0 software (Cyagen) was used to design p7(J4)-GFP and p7(H77)-GFP plasmids with a coding sequence containing codon optimized HCV p7 (J4 or H77) and eGFP sequences separated by a T2A linker sequence under control of an albumin promotor and its upstream enhancer sequence^51,92^. *E*. *coli* Stbl3 cultures cloned with the expression vectors (Cyagen) were cultured and plasmid DNA was purified using Endofree plasmid Giga kit (Quiagen) as per manufacturer’s instructions.

### Surrogate challenge

Transient transfection of liver cells was performed by hydrodynamic injection of plasmid DNA, as described elsewhere^93,94^. In brief, fully anesthetized mice received volumes of 1.6 ml PBS containing 0, 1, 10, 50 or 100 µg p7(J4)-GFP or p7(H77)-GFP plasmid injected into the tail vein within 6–10 seconds at a constant rate.

### Microscopy

Liver cells were fixed 15 minutes at 4 °C in Cytofix kit (BD Pharmingen) and washed twice in PBS followed by nuclei staining in Hoechst 33342 at 1:5000 in 10 minutes. 1–1.1E6 cells per well were allowed to settle overnight in 6-well plates at 4 °C and were visualized on a Zeiss AXIO observer Z1 system with minor adjustment of contrast in Zen 2 core v.2.4 software.

### Flow cytometry

For re-stimulation assays, cells were co-incubated with peptides (individually or as a pool of the 6 peptides spanning the p7 sequence; 2 µg/ml of each) and CD28/CD49d antibodies (clone 37.51 and 9C10; MFR4.B, BD Pharmingen) in media in 96-well plates for one hour at 37 °C followed by six hours incubation in the presence of 10 µg/ml brefeldin A. Cells were washed in FACS-buffer (PBS containing 1% FBS) and subsequently stained for surface markers (30 min at 4 °C) followed by washing, permeabilization using Cytofix/Cytoperm kit (BD Pharmingen) as per manufacturers protocol, and intracellular staining (30 min at 4 °C). For six-color flow cytometry-analysis anti-CD4-APC-Cy7 (clone GK1.5), anti-CD8-PerCP-Cy5.5 (clone 53–6.7) and anti-CD44-FITC (clone IM7) for surface staining were diluted 1:600 in FACS-buffer and anti-IFN-γ-PE-Cy7 (clone XMG1.2), anti-TNF-α-PE (clone MP6-XT22) and anti-IL-2-APC (clone JES6-5H4) for intracellular staining were diluted 1:200 in PermWash buffer (BD Pharmingen). Cells were subsequently washed and analyzed on a six-color FACSCanto instrument (BD Biosciences).

For 10-color flow cytometry-analysis, surface staining was performed with anti-CD4-BV510 (clone RM4-5, Biolegend), anti-CD8-PerCP-Cy5.5 and anti-CD44-Alexa700 (clone IM7, Biolegend) diluted 1:600, anti-CD3-BV650 (clone 17A2, Biolegend), anti-KLRG1-BV711 (clone 2F1, eBiosciences) and antiCD62L-FITC (clone MEL-14, BD Pharmingen) diluted 1:200 and anti-PD-1-PE-Cy7 (clone RMP1, Biolegend) diluted 1:100 in FACS-buffer. Intracellular IFN-γ, TNF-α, and IL-2 were stained with antibodies and concentrations identical to those used in the 6-color panel. FSC-A/SSC-A gating to identify lymphocytes was performed after doublet exclusion based on their FSC-A/FSC-H properties. These were further gated into CD3^+^ CD4^+^ or CD3^+^ CD8^+^ T cells followed by gating of CD4^+^ CD44^+^ and CD8^+^ CD44^+^ populations into IFN-γ, TNF-α and IL-2 producing T-cells. Boolean gates were applied to divide IFN-γ producing cells into three distinct subpopulations based on their ability to co-produce TNF-α alone or in combination with IL-2. Finally, Boolean gating was also employed to select antigen specific T-cell producing any cytokine, which were further gated into PD-1^hi^KLRG1^lo^ and PD-1^int^KLRG1^hi^ subsets analyzed for their expression level of IFN-γ, TNF-α, IL-2 and CD62L based on their median fluorescence intensity (MFI).

For analysis of CD107 expression, cells were prepared as described above, with the exception being anti-CD107a-FITC (clone 1D4B, BD Pharmingen), which was co-incubated during p7 (J4) peptide# 4 restimulation at a 1:100 dilution. CD8-APC-Cy7 (clone 53–6.7, BD Pharmingen), anti-IFN-γ-PerCP-Cy5.5 (clone XMG1.2, eBiosciences) and anti-TNF-α-allophycocyanin (clone MP6, BD Pharmingen) were all in 1:200 dilutions. Boolean gating was applied to CD8^+^ T-cells to determine the degree of co-production of IFN-γ and TNF-α by CD107^+^ cells.

GFP-expression of liver cells was performed on unstained cells fixed 15 minutes at 4 °C in Cytofix and washed twice in FACS-buffer before analysis on a BD Fortessa instrument. Large cells were gated based on their FSC-A/FSC-H profile excluding lymphocytes and were further gated into a FSC-A/SSC-A^hi^ profile enriched of hepatocytes^95^. GFP^+^ cells were identified based on their FITC/FSC-H profile. All flow cytometry data were analyzed using FlowJo software v10.1 (Tree Star Ashland).

### *In vivo* cytotoxicity

Splenocytes isolated from naïve C57BL/6 or BALB/c mice were divided into three fractions and labelled with CFSE at either 0.8 µM (CFSE^low^), 4 µM (CFSE^mid^) or 20 µM (CFSE^high^) in PBS at room temperature. After 10 minutes, ice-cold FBS was added to a final concentration of 30% and the cells were washed in full media. CFSE^low^ and CFSE^high^ fractions from C57BL/c mice were pulsed with 10 µg/ml p7 (J4) peptide #4 and #1, respectively. The CFSE^mid^ fraction was left unpulsed. The three fractions of cells were then mixed in 1:1:1 ratio, washed three times in RPMI 1640, and filtered through a 100 µm nylon mesh before they were injected into immunized C57BL/6 mice at volumes of 300 µl containing 3.3E7 cells/ml 12 days after the final vaccination. CFSE^low^ and CFSE^high^ fractions from BALB/c mice peptide #4 and #3, respectively, but otherwise treated identically as described above, before they were injected into vaccinated BALB/c mice. Eighteen hours after injection of the labelled cells, splenocytes were recovered from mice and labelled with anti-MHC-II-PerCP-Cy5.5 (BioLegend, clone M5/114.15.2) and analyzed on flow cytometry. Cells were gated based on their FSC-A/FSC-H properties and the three cell fractions could be distinguished based on their CFSE profile. Lysis of peptide-pulsed target cells relative to unpulsed cells was determined using the equation: 1 − ((% peptide pulsed cells^vaccinated mice^/% unpulsed cells^vaccinated mice^) − (% peptide pulsed cells^control mice^/% unpulsed cells^control mice^)).

### *In silico* epitope predictions of CD8^+^ T-cell epitopes

CD8^+^ T-cell epitopes within p7 peptide #4 derived from the HCV J4 or H77 sequences were performed using the T-cell epitope processing and prediction tool provided by the Immune Epitope Database (IEDB) Analysis Resource (http://tools.iedb.org/processing/) employing the NetMHCpan 3.0 algorithm.

### Statistical analysis

Analysis of variance (ANOVA) followed by Tukey´s posttest was used in all assays to compare differences between multiple vaccine groups. Statistically significant differences are marked with asterisks in figures and explained in the figure legends. All statistical analysis was performed using Prism 6 software (GraphPad).

## Supplementary information

Supplementary Figures S1-S8 and Table S1

## Data Availability

All data generated or analysed during this study are included in this published article and its Supplementory Information files.

## References

[1] Cox AL (2015). MEDICINE. Global control of hepatitis C virus. Science (New York, N.Y.).

[2] Walker CM, Grakoui A (2015). Hepatitis C virus: why do we need a vaccine to prevent a curable persistent infection?. Current opinion in immunology.

[3] Bartenschlager R (2018). Critical challenges and emerging opportunities in hepatitis C virus research in an era of potent antiviral therapy: Considerations for scientists and funding agencies. Virus research.

[4] Forns X, Bukh J, Purcell RH (2002). The challenge of developing a vaccine against hepatitis C virus. Journal of hepatology.

[5] Bukh J (2012). Animal models for the study of hepatitis C virus infection and related liver disease. Gastroenterology.

[6] Forns X, Purcell RH, Bukh J (1999). Quasispecies in viral persistence and pathogenesis of hepatitis C virus. Trends in microbiology.

[7] Farci P, Bukh J, Purcell RH (1997). The quasispecies of hepatitis C virus and the host immune response. Springer seminars in immunopathology.

[8] Islam N (2017). Hepatitis C cross-genotype immunity and implications for vaccine development. Scientific reports.

[9] Smith DB (2014). Expanded classification of hepatitis C virus into 7 genotypes and 67 subtypes: updated criteria and genotype assignment web resource. Hepatology (Baltimore, Md.).

[10] Christiansen D (2018). Immunological responses following administration of a genotype 1a/1b/2/3a quadrivalent HCV VLP vaccine. Scientific reports.

[11] Wijesundara Danushka K., Gummow Jason, Li Yanrui, Yu Wenbo, Quah Benjamin J., Ranasinghe Charani, Torresi Joseph, Gowans Eric J., Grubor-Bauk Branka (2018). Induction of Genotype Cross-Reactive, Hepatitis C Virus-Specific, Cell-Mediated Immunity in DNA-Vaccinated Mice. Journal of Virology.

[12] Bukh J (2016). The history of hepatitis C virus (HCV): Basic research reveals unique features in phylogeny, evolution and the viral life cycle with new perspectives for epidemic control. Journal of hepatology.

[13] Walker CM (2017). Designing an HCV vaccine: a unique convergence of prevention and therapy?. Current opinion in virology.

[14] Panagioti E, Klenerman P, Lee LN, van der Burg SH, Arens R (2018). Features of Effective T Cell-Inducing Vaccines against Chronic Viral Infections. Frontiers in immunology.

[15] Bukh J (2008). Previously infected chimpanzees are not consistently protected against reinfection or persistent infection after reexposure to the identical hepatitis C virus strain. Journal of virology.

[16] Meunier JC (2008). Isolation and characterization of broadly neutralizing human monoclonal antibodies to the e1 glycoprotein of hepatitis C virus. Journal of virology.

[17] Lucas M (2018). Evidence of CD4(+) T cell-mediated immune pressure on the Hepatitis C virus genome. Scientific reports.

[18] Woollard D (2003). Characterization of HCV-specific Patr class II restricted CD4+ T cell responses in an acutely infected chimpanzee. Hepatology.

[19] Ciuffreda D (2008). Polyfunctional HCV-specific T-cell responses are associated with effective control of HCV replication. European journal of immunology.

[20] Schulze ZJ (2012). Broadly directed virus-specific CD4 + T cell responses are primed during acute hepatitis C infection, but rapidly disappear from human blood with viral persistence. The Journal of experimental medicine.

[21] Thimme R (2001). Determinants of viral clearance and persistence during acute hepatitis C virus infection. The Journal of experimental medicine.

[22] Schulze zur Wiesch J (2005). Broad repertoire of the CD4+ Th cell response in spontaneously controlled hepatitis C virus infection includes dominant and highly promiscuous epitopes. Journal of immunology (Baltimore, Md.: 1950).

[23] Mikkelsen M, Bukh J (2007). Current status of a hepatitis C vaccine: encouraging results but significant challenges ahead. Current infectious disease reports.

[24] Sumpter TL, Abe M, Tokita D, Thomson AW (2007). Dendritic cells, the liver, and transplantation. Hepatology (Baltimore, Md.).

[25] Breous E, Somanathan S, Vandenberghe LH, Wilson JM (2009). Hepatic regulatory T cells and Kupffer cells are crucial mediators of systemic T cell tolerance to antigens targeting murine liver. Hepatology (Baltimore, Md.).

[26] Grakoui A, Crispe IN (2016). Presentation of hepatocellular antigens. Cellular & molecular immunology.

[27] Lang Kuhs KA (2011). Peripheral immunization induces functional intrahepatic hepatitis C specific immunity following selective retention of vaccine-specific CD8 T cells by the liver. Human vaccines.

[28] Gola Anita, Silman Daniel, Walters Adam A., Sridhar Saranya, Uderhardt Stefan, Salman Ahmed M., Halbroth Benedict R., Bellamy Duncan, Bowyer Georgina, Powlson Jonathan, Baker Megan, Venkatraman Navin, Poulton Ian, Berrie Eleanor, Roberts Rachel, Lawrie Alison M., Angus Brian, Khan Shahid M., Janse Chris J., Ewer Katie J., Germain Ronald N., Spencer Alexandra J., Hill Adrian V. S. (2018). Prime and target immunization protects against liver-stage malaria in mice. Science Translational Medicine.

[29] Barnes E (2012). Novel adenovirus-based vaccines induce broad and sustained T cell responses to HCV in man. Science translational medicine.

[30] Swadling L (2014). A human vaccine strategy based on chimpanzee adenoviral and MVA vectors that primes, boosts, and sustains functional HCV-specific T cell memory. Science translational medicine.

[31] Knudsen NP (2016). Different human vaccine adjuvants promote distinct antigen-independent immunological signatures tailored to different pathogens. Scientific reports.

[32] Obara W (2018). Present status and future perspective of peptide-based vaccine therapy for urological cancer. Cancer science.

[33] Korsholm KS (2014). Induction of CD8 + T-cell responses against subunit antigens by the novel cationic liposomal CAF09 adjuvant. Vaccine.

[34] Schmidt ST (2016). The administration route is decisive for the ability of the vaccine adjuvant CAF09 to induce antigen-specific CD8( + ) T-cell responses: The immunological consequences of the biodistribution profile. Journal of controlled release: official journal of the Controlled Release Society.

[35] Schmidt ST (2018). Induction of Cytotoxic T-Lymphocyte Responses Upon Subcutaneous Administration of a Subunit Vaccine Adjuvanted With an Emulsion Containing the Toll-Like. Receptor 3 Ligand Poly(I:C). Frontiers in immunology.

[36] Liang TJ (2013). Current progress in development of hepatitis C virus vaccines. Nature medicine.

[37] Bailey Justin R., Barnes Eleanor, Cox Andrea L. (2019). Approaches, Progress, and Challenges to Hepatitis C Vaccine Development. Gastroenterology.

[38] Griffin SD (2003). The p7 protein of hepatitis C virus forms an ion channel that is blocked by the antiviral drug, Amantadine. FEBS letters.

[39] Steinmann E (2007). Hepatitis C virus p7 protein is crucial for assembly and release of infectious virions. PLoS pathogens.

[40] Pavlovic D (2003). The hepatitis C virus p7 protein forms an ion channel that is inhibited by long-alkyl-chain iminosugar derivatives. Proceedings of the National Academy of Sciences of the United States of America.

[41] Callendret B (2011). Transmission of clonal hepatitis C virus genomes reveals the dominant but transitory role of CD8(+) T cells in early viral evolution. Journal of virology.

[42] Sakai A (2003). The p7 polypeptide of hepatitis C virus is critical for infectivity and contains functionally important genotype-specific sequences. Proceedings of the National Academy of Sciences of the United States of America.

[43] Filskov, J. *et al*. Broadening CD4 + and CD8 + T Cell Responses against Hepatitis C Virus by Vaccination with NS3 Overlapping Peptide Panels in Cross-Priming Liposomes. *Journal of virology* **91**, 10.1128/jvi.00130-17 (2017). 10.1128/JVI.00130-17 PMC5487548 28446674

[44] Yanagi M (1998). Transcripts of a chimeric cDNA clone of hepatitis C virus genotype 1b are infectious *in vivo*. Virology.

[45] Hansen SG (2009). Effector memory T cell responses are associated with protection of rhesus monkeys from mucosal simian immunodeficiency virus challenge. Nature medicine.

[46] Bukh J, Forns X, Emerson SU, Purcell RH (2001). Studies of hepatitis C virus in chimpanzees and their importance for vaccine development. Intervirology.

[47] Ahlen G (2005). *In vivo* clearance of hepatitis C virus nonstructural 3/4A-expressing hepatocytes by DNA vaccine-primed cytotoxic T lymphocytes. The Journal of infectious diseases.

[48] Yu W, Grubor-Bauk B, Mullick R, Das S, Gowans EJ (2014). Immunocompetent mouse models to evaluate intrahepatic T cell responses to HCV vaccines. Human vaccines & immunotherapeutics.

[49] Yu W, Grubor-Bauk B, Gargett T, Garrod T, Gowans EJ (2014). A novel challenge model to evaluate the efficacy of hepatitis C virus vaccines in mice. Vaccine.

[50] Ahlen G (2009). Cleavage of the IPS-1/Cardif/MAVS/VISA does not inhibit T cell-mediated elimination of hepatitis C virus non-structural 3/4A-expressing hepatocytes. Gut.

[51] Izban MG, Papaconstantinou J (1989). Cell-specific expression of mouse albumin promoter. Evidence for cell-specific DNA elements within the proximal promoter region and cis-acting DNA elements upstream of -160. The Journal of biological chemistry.

[52] Ryan MD, King AM, Thomas GP (1991). Cleavage of foot-and-mouth disease virus polyprotein is mediated by residues located within a 19 amino acid sequence. The Journal of general virology.

[53] Liu Z (2017). Systematic comparison of 2A peptides for cloning multi-genes in a polycistronic vector. Scientific reports.

[54] Reiley WW (2010). Distinct functions of antigen-specific CD4 T cells during murine Mycobacterium tuberculosis infection. Proceedings of the National Academy of Sciences of the United States of America.

[55] Shuck K, Lamb RA, Pinto LH (2000). Analysis of the pore structure of the influenza A virus M(2) ion channel by the substituted-cysteine accessibility method. Journal of virology.

[56] Park SH (2003). Three-dimensional structure of the channel-forming trans-membrane domain of virus protein “u” (Vpu) from HIV-1. Journal of molecular biology.

[57] Sakai A (2007). *In vivo* study of the HC-TN strain of hepatitis C virus recovered from a patient with fulminant hepatitis: RNA transcripts of a molecular clone (pHC-TN) are infectious in chimpanzees but not in Huh7.5 cells. Journal of virology.

[58] Lauer GM (2004). High resolution analysis of cellular immune responses in resolved and persistent hepatitis C virus infection. Gastroenterology.

[59] Korsholm KS (2013). Broadening of the T-cell repertoire to HIV-1 Gag p24 by vaccination of HLA-A2/DR transgenic mice with overlapping peptides in the CAF05 adjuvant. PloS one.

[60] Aagaard CS, Hoang TT, Vingsbo-Lundberg C, Dietrich J, Andersen P (2009). Quality and vaccine efficacy of CD4+ T cell responses directed to dominant and subdominant epitopes in ESAT-6 from Mycobacterium tuberculosis. Journal of immunology (Baltimore, Md.: 1950).

[61] Woodworth JS (2014). Protective CD4 T cells targeting cryptic epitopes of Mycobacterium tuberculosis resist infection-driven terminal differentiation. Journal of immunology (Baltimore, Md.: 1950).

[62] Olsen, A. W., Hansen, P. R., Holm, A. & Andersen, P. Efficient protection against Mycobacterium tuberculosis by vaccination with a single subdominant epitope from the ESAT-6 antigen. *European journal of immunology* **30**, 1724–1732, 10.1002/1521-4141(200006)30:6<1724::aid-immu1724>3.0.co;2-a (2000). 10.1002/1521-4141(200006)30:6<1724::AID-IMMU1724>3.0.CO;2-A 10898510

[63] Zwaveling S (2002). Established human papillomavirus type 16-expressing tumors are effectively eradicated following vaccination with long peptides. Journal of immunology (Baltimore, Md.: 1950).

[64] Fayolle C, Deriaud E, Leclerc C (1991). *In vivo* induction of cytotoxic T cell response by a free synthetic peptide requires CD4+ T cell help. Journal of immunology (Baltimore, Md.: 1950).

[65] Yellen-Shaw AJ, Wherry EJ, Dubois GC, Eisenlohr LC (1997). Point mutation flanking a CTL epitope ablates *in vitro* and *in vivo* recognition of a full-length viral protein. Journal of immunology (Baltimore, Md.: 1950).

[66] Shastri N, Serwold T, Gonzalez F (1995). Presentation of endogenous peptide/MHC class I complexes is profoundly influenced by specific C-terminal flanking residues. Journal of immunology (Baltimore, Md.: 1950).

[67] Lindenstrom T, Aagaard C, Christensen D, Agger EM, Andersen P (2014). High-frequency vaccine-induced CD8(+) T cells specific for an epitope naturally processed during infection with Mycobacterium tuberculosis do not confer protection. European journal of immunology.

[68] Wahid R, Cannon MJ, Chow M (2005). Virus-specific CD4+ and CD8+ cytotoxic T-cell responses and long-term T-cell memory in individuals vaccinated against polio. Journal of virology.

[69] Soghoian DZ (2012). HIV-specific cytolytic CD4 T cell responses during acute HIV infection predict disease outcome. Science translational medicine.

[70] Brown DM, Lee S, Garcia-Hernandez Mde L, Swain SL (2012). Multifunctional CD4 cells expressing gamma interferon and perforin mediate protection against lethal influenza virus infection. Journal of virology.

[71] Brown DM, Dilzer AM, Meents DL, Swain SL (2006). CD4 T cell-mediated protection from lethal influenza: perforin and antibody-mediated mechanisms give a one-two punch. Journal of immunology (Baltimore, Md.: 1950).

[72] Aslan N (2006). Cytotoxic CD4 T cells in viral hepatitis. Journal of viral hepatitis.

[73] Brown DM, Kamperschroer C, Dilzer AM, Roberts DM, Swain SL (2009). IL-2 and antigen dose differentially regulate perforin- and FasL-mediated cytolytic activity in antigen specific CD4+ T cells. Cellular immunology.

[74] Lu Z (2000). CD40-independent pathways of T cell help for priming of CD8(+) cytotoxic T lymphocytes. The Journal of experimental medicine.

[75] Dienes HP, Hutteroth T, Hess G, Meuer SC (1987). Immunoelectron microscopic observations on the inflammatory infiltrates and HLA antigens in hepatitis B and non-A, non-B. Hepatology (Baltimore, Md.).

[76] Franco A (1988). Expression of class I and class II major histocompatibility complex antigens on human hepatocytes. Hepatology (Baltimore, Md.).

[77] Paterson AC (1988). HLA expression in human hepatocellular carcinoma. British journal of cancer.

[78] Doumba PP, Nikolopoulou M, Gomatos IP, Konstadoulakis MM, Koskinas J (2013). Co-culture of primary human tumor hepatocytes from patients with hepatocellular carcinoma with autologous peripheral blood mononuclear cells: study of their *in vitro* immunological interactions. BMC gastroenterology.

[79] Sung CH (1989). Expression of class I and class II major histocompatibility antigens on human hepatocellular carcinoma. The Journal of clinical investigation.

[80] Paroli M (1994). Human hepatoma cells expressing MHC antigens display accessory cell function: dependence on LFA-1/ICAM-1 interaction. Immunology.

[81] Burghardt S (2013). Hepatocytes contribute to immune regulation in the liver by activation of the Notch signaling pathway in T cells. Journal of immunology (Baltimore, Md.: 1950).

[82] Frauwirth K, Shastri N (2001). Introducing endogenous antigens into the major histocompatibility complex (MHC) class II presentation pathway. Both Ii mediated inhibition and enhancement of endogenous peptide/MHC class II presentation require the same Ii domains. Immunology.

[83] Herkel J (2003). MHC class II-expressing hepatocytes function as antigen-presenting cells and activate specific CD4 T lymphocyutes. Hepatology (Baltimore, Md.).

[84] Pal S, Tifrea DF, Follmann F, Andersen P, de la Maza LM (2017). The cationic liposomal adjuvants CAF01 and CAF09 formulated with the major outer membrane protein elicit robust protection in mice against a Chlamydia muridarum respiratory challenge. Vaccine.

[85] Thakur A (2018). Targeting the Mincle and TLR3 receptor using the dual agonist cationic adjuvant formulation 9 (CAF09) induces humoral and polyfunctional memory T cell responses in calves. PloS one.

[86] Weiner GJ (2015). Building better monoclonal antibody-based therapeutics. Nature reviews. Cancer.

[87] Forthal DN, Gilbert PB, Landucci G, Phan T (2007). Recombinant gp120 vaccine-induced antibodies inhibit clinical strains of HIV-1 in the presence of Fc receptor-bearing effector cells and correlate inversely with HIV infection rate. Journal of immunology (Baltimore, Md.: 1950).

[88] Billerbeck E (2017). Mouse models of acute and chronic hepacivirus infection. Science (New York, N.Y.).

[89] Trivedi Sheetal, Murthy Satyapramod, Sharma Himanshu, Hartlage Alex S., Kumar Arvind, Gadi Sashi V., Simmonds Peter, Chauhan Lokendra V., Scheel Troels K.H., Billerbeck Eva, Burbelo Peter D., Rice Charles M., Lipkin W. Ian, Vandegrift Kurt, Cullen John M., Kapoor Amit (2018). Viral persistence, liver disease, and host response in a hepatitis C-like virus rat model. Hepatology.

[90] Bukh J (2010). Challenge pools of hepatitis C virus genotypes 1-6 prototype strains: replication fitness and pathogenicity in chimpanzees and human liver-chimeric mouse models. The Journal of infectious diseases.

[91] Kolykhalov AA (1997). Transmission of hepatitis C by intrahepatic inoculation with transcribed RNA. Science (New York, N.Y.).

[92] Pinkert CA, Ornitz DM, Brinster RL, Palmiter RD (1987). An albumin enhancer located 10 kb upstream functions along with its promoter to direct efficient, liver-specific expression in transgenic mice. Genes & development.

[93] Liu F, Song Y, Liu D (1999). Hydrodynamics-based transfection in animals by systemic administration of plasmid DNA. Gene therapy.

[94] Zhang G (2004). Hydroporation as the mechanism of hydrodynamic delivery. Gene therapy.

[95] Goncalves LA, Vigario AM, Penha-Goncalves C (2007). Improved isolation of murine hepatocytes for *in vitro* malaria liver stage studies. Malaria journal.

