# Supplementary material for: HCV p7 as a novel vaccine-target inducing multifunctional CD4+ and CD8+ T-cells targeting liver cells expressing the viral antigen
Source: Sci Rep. 2019 Oct 1;9:14085. doi: 10.1038/s41598-019-50365-z (PMC6773770; doi:10.1038/s41598-019-50365-z)
Supplement: Supplementary file 1 — Supplementary Figures S1-S8 and Table S1 [file 41598_2019_50365_MOESM1_ESM.pdf]

Supplementary Figures S1-8 and Table S1 for the manuscript entitled

"HCV p7 as a novel vaccine-target inducing multifunctional $CD4^+$ and $CD8^+$ T-cells targeting liver cells expressing the viral antigen"

Author list:
Jonathan Filskov, Peter Andersen, Else Marie Agger, Jens Bukh

Supplementary Figure S1

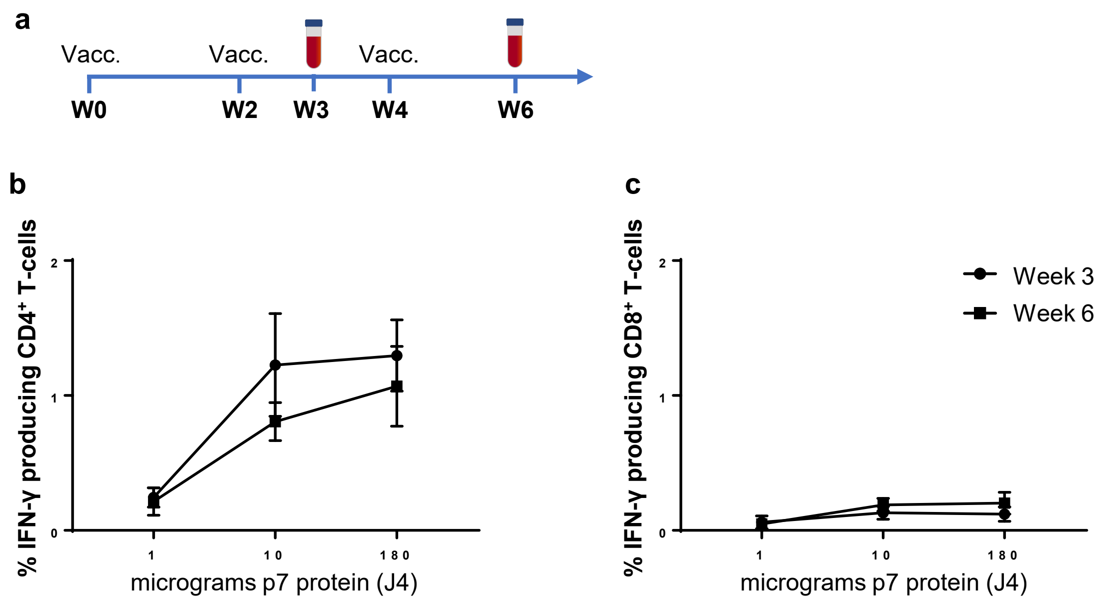

Supplementary Figure S1

High antigen dose does not increase immunogenicity of the HCV p7 protein vaccine. CB6F1 mice were vaccinated three times at two-week intervals with 1 μg, 10 μg or 180 μg of the HCV p7 protein (strain J4). (**a**) As illustrated on the time-line, PBMCs were harvested from individual mice three or six weeks after the priming vaccination and were restimulated with a pool of overlapping p7 (J4) peptides followed by FACS-analysis. Frequencies of antigen-specific IFN-γ producing $CD44^+$ $CD4^+$ T-cells (**b**) or $CD44^+$ $CD8^+$ T-cells (**c**) are shown as means and SEM on graphs (n = 5 to 6 mice in each vaccine group).

Supplementary Figure S2

a

<u>p7 pepmix (H77)</u>

Pept#1: ALENLVILNAASLAGTHGLV
Pept#2: ASLAGTHGLVSFLVFFCFAW
Pept#3: SFLVFFCFAWYLKGRWVPGA
Pept#4: YLKGRWVPGAVYAFYGMWPL
Pept#5: VYAFYGMWPLLLLLLALPQR
Pept#6: LLLLLALPQRAYA

b

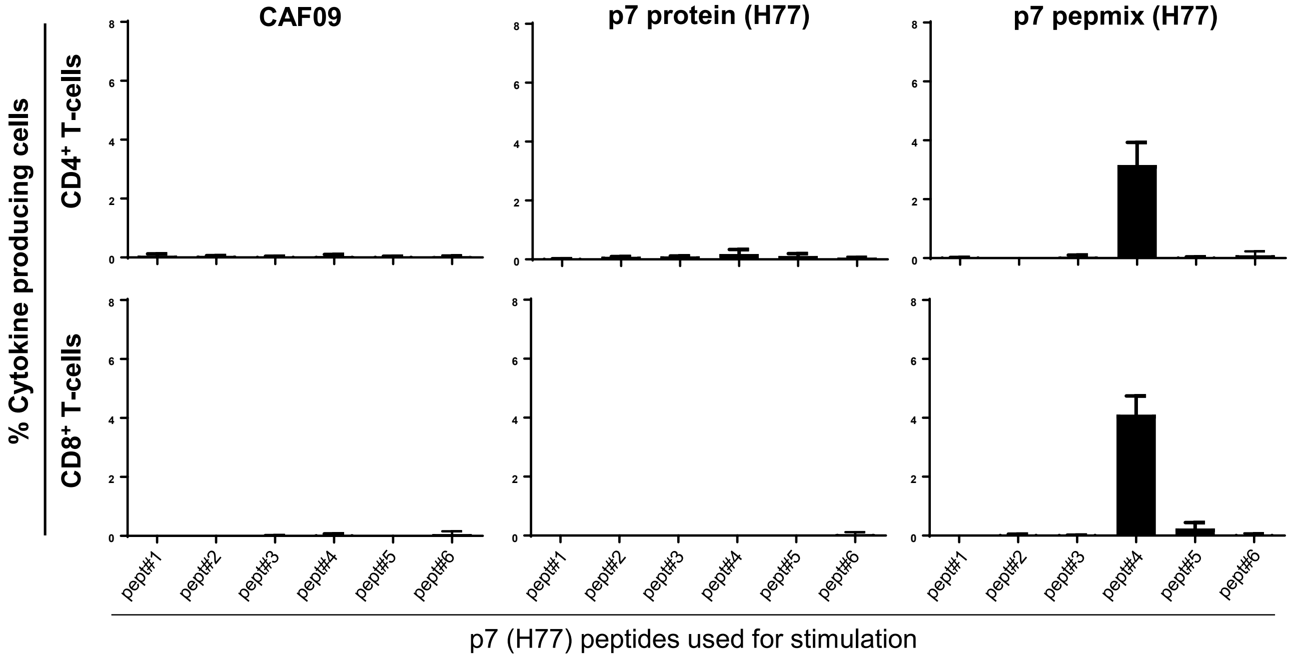

<u>Supplementary Figure S2</u>

Repertoires of p7 pepmix (H77) specific T-cells.

(**a**) The amino acid sequence of peptides comprising the p7 pepmix (H77) are shown. (**b**) Mice were vaccinated three times at 2-week intervals with the CAF09 adjuvant alone, HCV p7 protein or pepmix (H77) as indicated above the graphs. Splenocytes were isolated and pooled for each vaccine group and subsequently restimulated with each of the individual peptides (pept#1 to pept#6, indicated below the *x*-axis), to map the repertoire of epitope-specific responses. Bars show the total frequency of epitope-specific $CD44^+$ $CD4^+$ T-cells (upper panels) and $CD44^+$ $CD8^+$ T-cells (lower panels) able to produce IFN-γ, TNF-α or IL-2 in any combination. Data are shown as means and SEM from two independent experiments where splenocytes were pooled from 4-5 mice in each vaccine group.

Supplementary Figure S3

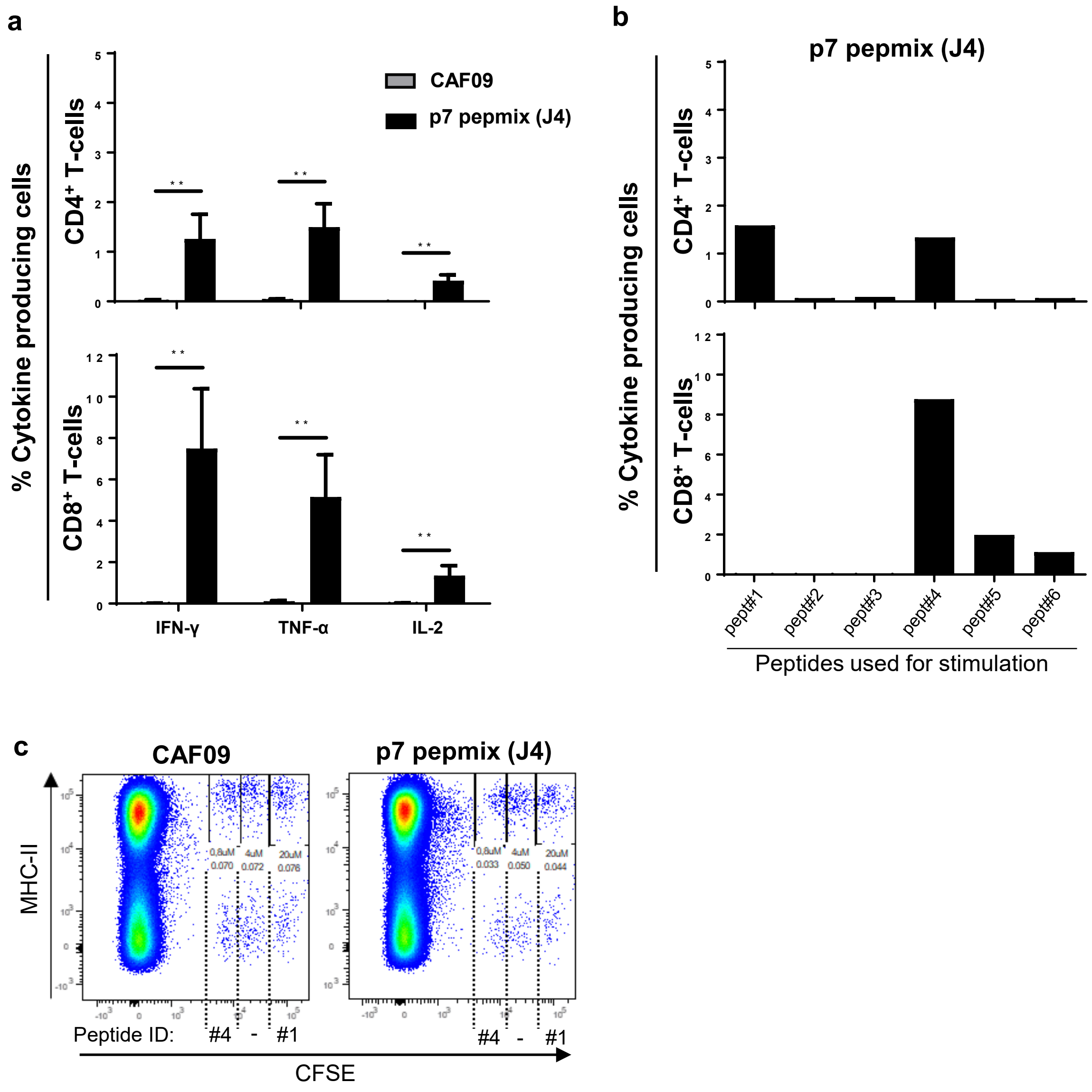

<u>Supplementary Figure S3</u>

Cytotoxic potential of vaccine-induced T-cells in C57BL/6 mice. (**a**) Splenocytes from individual mice vaccinated with HCV p7 pepmix (J4) were isolated and restimulated with a pool of overlapping p7 (J4) peptides. Frequencies of antigen-specific IFN-γ, TNF-α and IL-2 producing $CD44^+$ $CD4^+$ T-cells (upper panel) and $CD44^+$ $CD8^+$ T-cells (lower panel) out of the total $CD4^+$ or $CD8^+$ T-cell population respectively, is shown in the bar charts as means and standard errors of the mean (SEM) (n = 6 mice in each vaccine group). $^{**}P < 0.01$. (**b**) Splenocytes from p7 pepmix (J4) vaccinated mice were pooled (n = 6 mice) and restimulated with each of the individual p7 peptides to map the repertoire of epitope-specific $CD44^+$ $CD4^+$ T-cells (upper panel) and $CD44^+$ $CD8^+$ T-cells (lower panel) able to produce IFN-γ, TNF-α or IL-2 in any combination. (**c**) CFSE-labelled target cells from naïve mice were pulsed with single p7 peptides #1 or #4 or left untouched and subsequently injected i.v. into CAF09 and p7 pepmix (J4) vaccinated mice 12 days after the final immunization as indicated on the timeline in Fig. 2c. Eighteen hours after i.v. injection, splenocytes were isolated from individual mice and analyzed by FACS to determine their CFSE fluorescence. Representative plots of the three target cell populations in controls (left panel) and p7 pepmix vaccinated mice (right panel) were identified based on their CFSE-fluorescence and MHC-II expression (n = 5 mice in each vaccine group).

Supplementary Figure S4

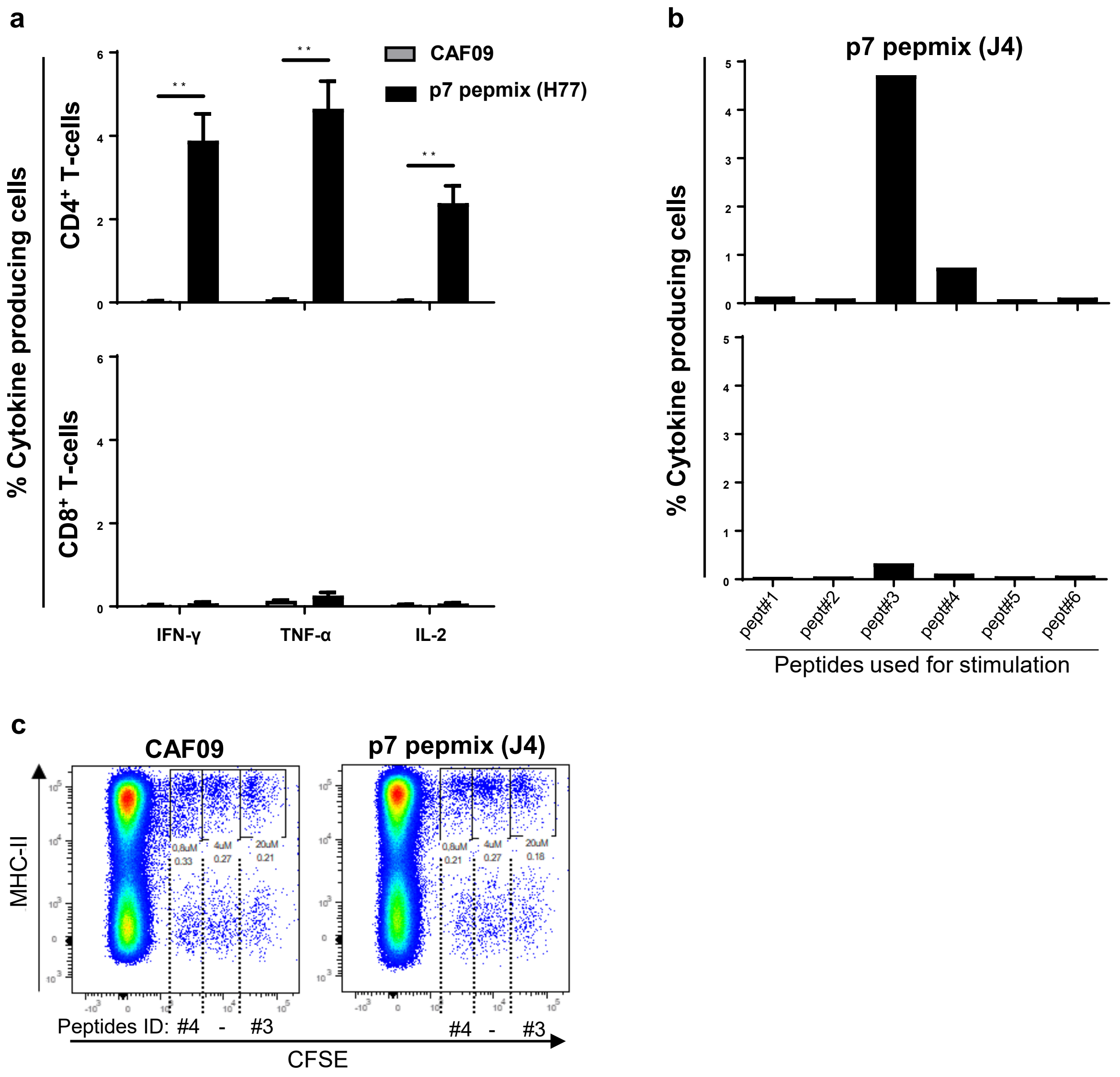

<u>Supplementary Figure S4</u>

Specific killing by cytotoxic CD4+ T cells in BALB/c mice

BALB/c mice were vaccinated three times at 2-week intervals with p7 pepmix (J4). (**a**) Splenocytes from individual mice were isolated and restimulated with a pool of overlapping p7 (J4) peptides and frequencies of antigen-specific, cytokine producing $CD44^+$ $CD4^+$ T-cells (upper panel) and $CD44^+$ $CD8^+$ T-cells (lower panel) is shown in the bar charts as means and standard errors of the mean (SEM) (n = 5 to 6 mice in each vaccine group). $^{**}P < 0.01$. (**b**) Splenocytes from p7 pepmix (J4) vaccinated mice (n = 5 mice) were pooled restimulated with individual p7 peptides to map epitope-specific $CD44^+$ $CD4^+$ T-cells (upper panel) and $CD44^+$ $CD8^+$ T-cells (lower panel). (**c**) CFSE-labelled target cells from naïve mice were pulsed with the single p7 peptides #3 or #4 or left untouched and subsequently injected i.v. into CAF09 and p7 pepmix (J4) vaccinated mice 12 days after the final immunization. Eighteen hours after i.v. injection, splenocytes were isolated from individual mice and the three target cell populations were identified based on their CFSE- and MHC-II profile. The plots are representative for 5 mice in each vaccine group.

Supplementary Figure S5

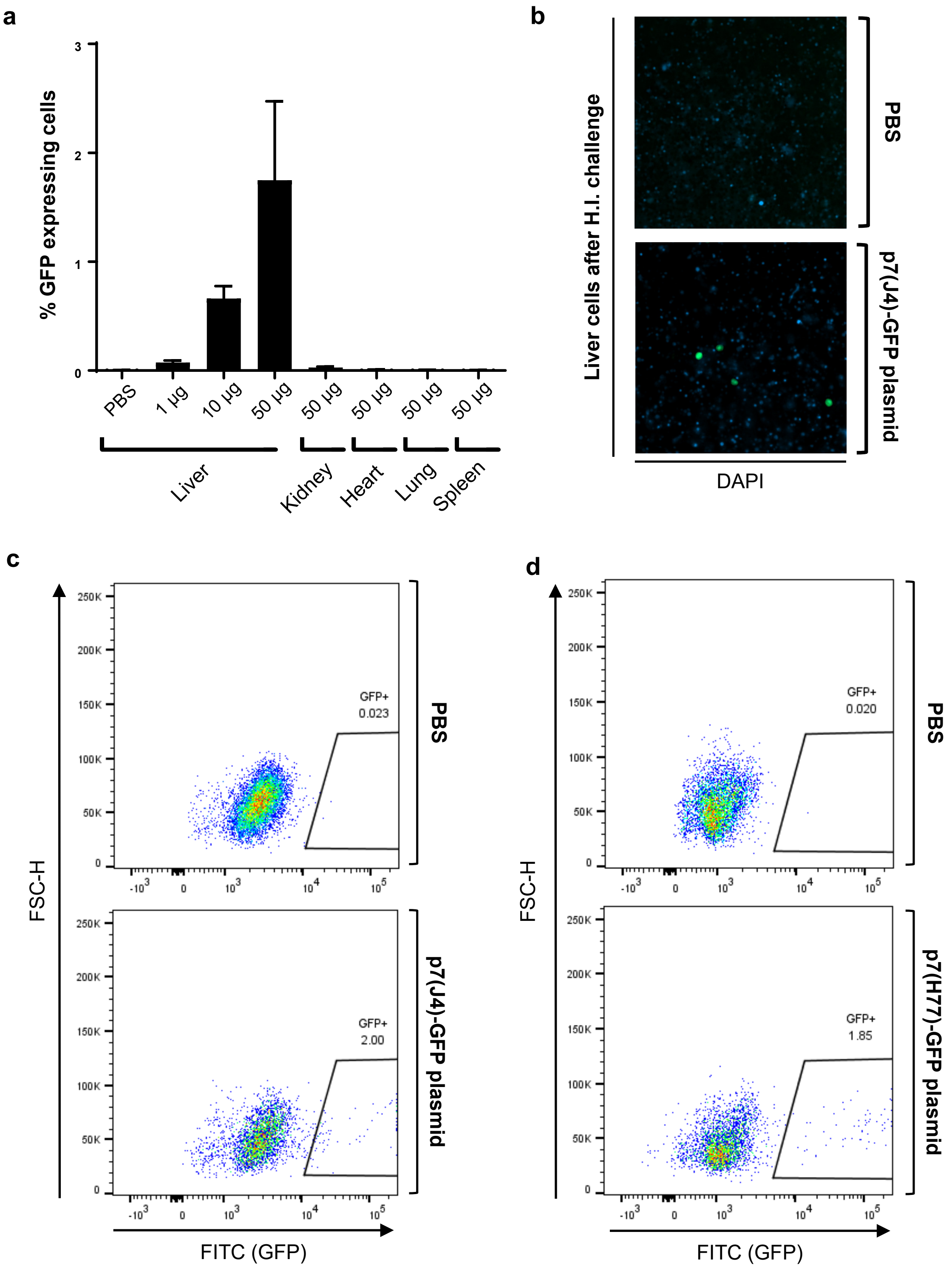

Supplementary Figure S5. Surrogate challenge model based on liver specific expression of HCV p7 and GFP in naïve mice. (**a**) GFP-expressing liver cells from mice challenged with 1, 10 or 50 µg of p7(J4)-GFP plasmid, three days after hydrodynamic injection, were identified by FACS-analysis. Mice injected with 50 µg plasmid were also assessed for GFP-expressing cells in the kidney, heart, lung and spleen. Means and SEM are indicated on the graph (n = 5 to 6 mice per group). (**b**) DAPI-stained liver cells isolated from mice 4 days after hydrodynamic injection (H.I.) with PBS or p7(J4)-GFP plasmid were visualized by fluorescence microscopy to assess GFP-expression. (**c**) Representative FACS-plots showing gating of FITC-positives to identify GFP-expressing liver cells 4 days after injection with PBS (upper panel) or 100 µg p7(J4)-GFP plasmid (lower panel). (**d**) Similarly, GFP-expressing liver cells could be identified after hydrodynamic injection of plasmid DNA expressing strain H77 (genotype 1a) p7 and GFP, p7(H77)-GFP.

Supplementary Figure S6

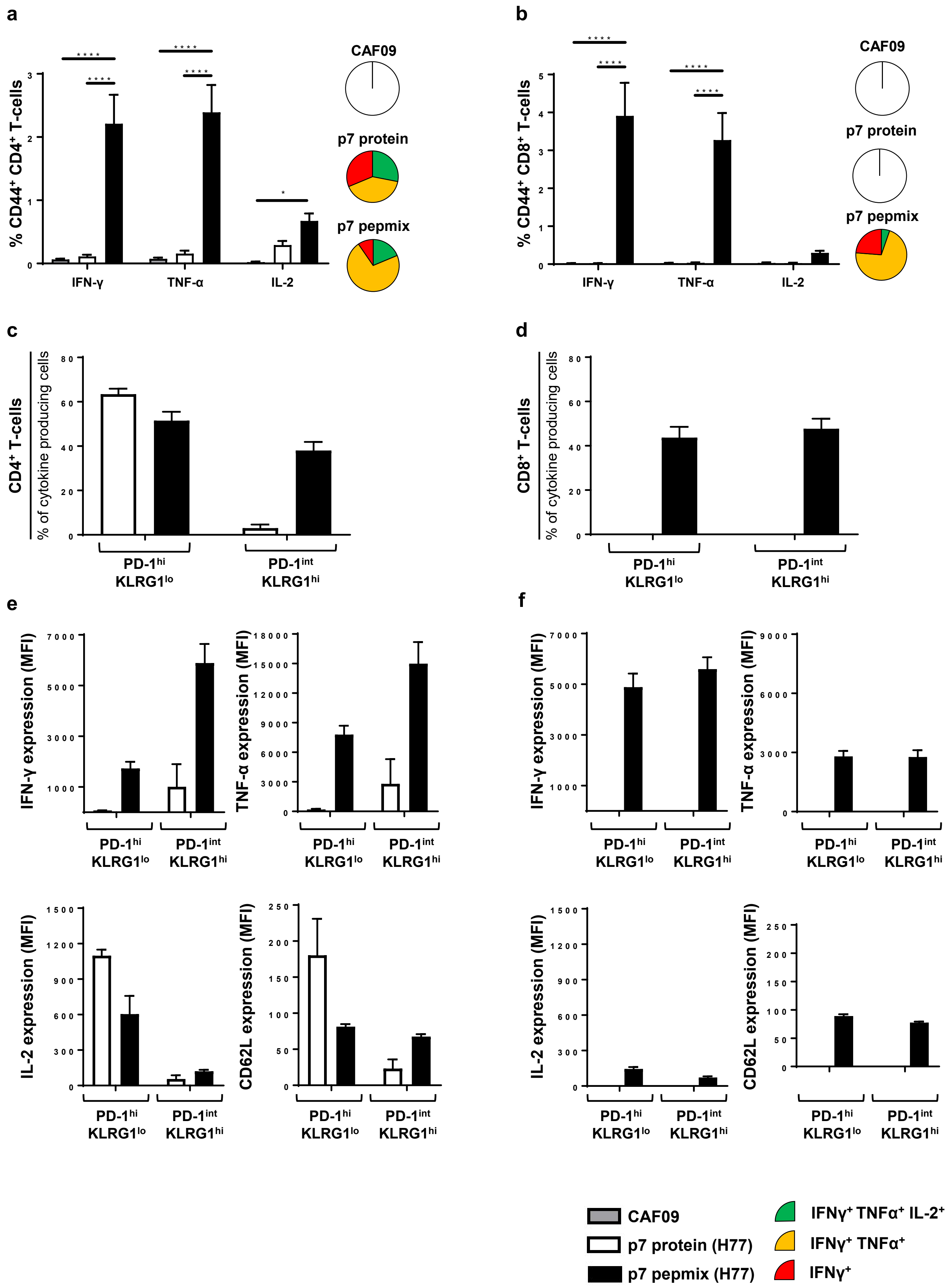

Supplementary Figure S6

Pre-challenge cytokine- and phenotypic characterization of T-cell subsets in vaccinated mice. PBMCs purified from mice ten days after the final vaccination and immune responses were evaluated similar to post-challenge responses. Bars indicate frequencies of IFN-γ, TNF-α and IL-2 producing $CD44^+$ $CD4^+$ T-cells (**a**) or $CD44^+$ $CD8^+$ T-cells (**b**) and pie charts show the distribution of IFN-γ positives co-producing TNF-α alone or in combination with IL-2. The frequencies of p7-specific $PD\text{-}1^{hi}/KLRG1^{lo}$ and $PD\text{-}1^{int}/KLRG1^{hi}$ $CD44^+$ $CD4^+$ T-cells (**c**) and $CD44^+$ $CD8^+$ T-cells (**d**) is shown on graphs. Further analysis shows the relative expression (MFI) of IFN-γ, TNF-α, IL-2 and CD62L by these $CD44^+$ $CD4^+$ T-cells (**e**) and $CD44^+$ $CD8^+$ T-cells (**f**). Bars represent means and standard errors of the mean (SEM) (n = 7 to 12 mice in each vaccine group). *, $P < 0.05$; ****$P < 0.0001$.

Supplementary Figure S7

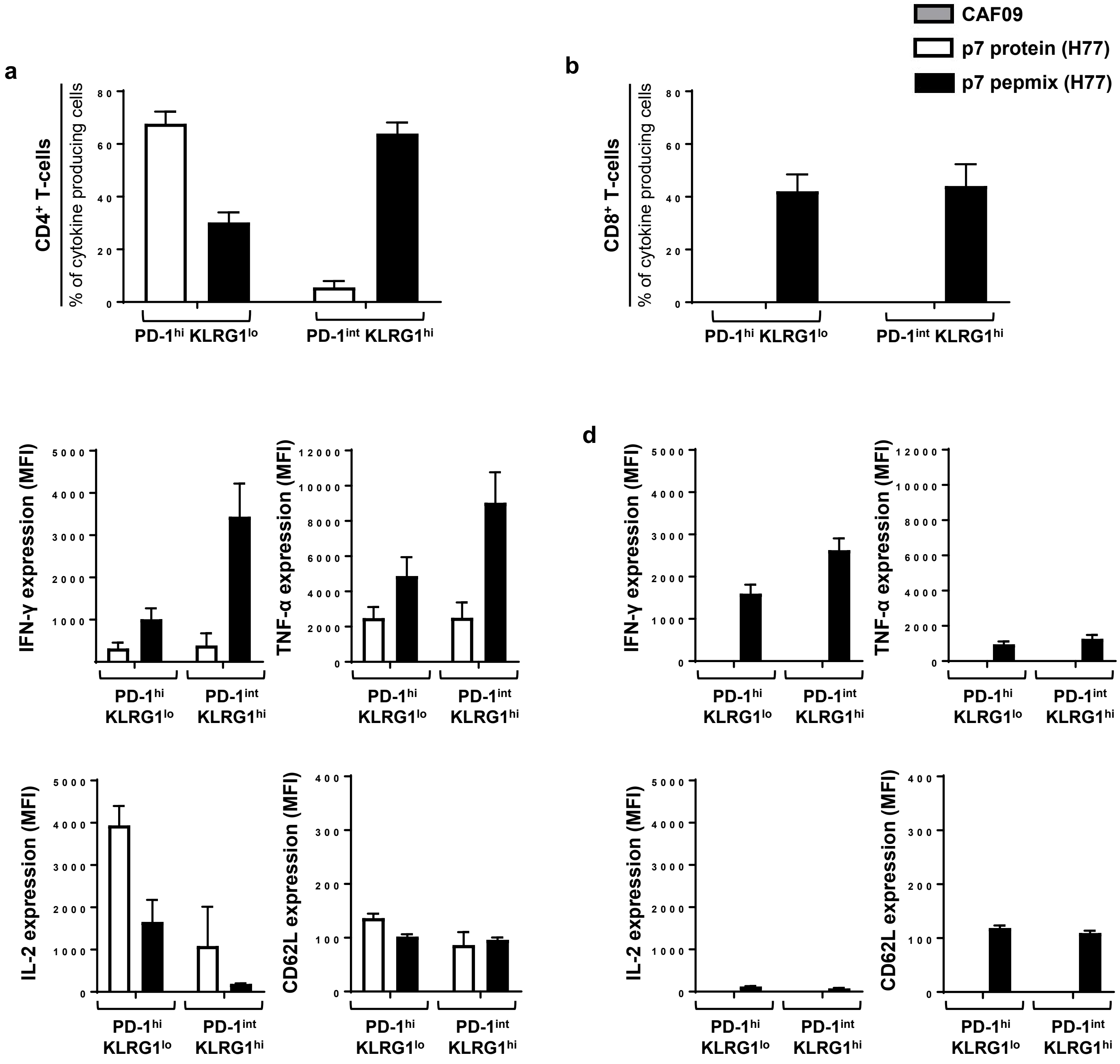

Supplementary Figure S7

Post-challenge characterization of T-cell subsets in vaccinated mice. Cytokine producing PBMCs purified from mice five days after challenge with p7(H77)-GFP plasmid (see Fig. 5a and 5b) were gated into gated into PD-1^{hi}/KLRG1^{lo} and PD-1^{int}/KLRG1^{hi} T-cell subpopulations and assessed for their relative expression (MFI) of IFN-γ, TNF-α, IL-2 and CD62L (**a** and **c**, CD4^{+} T-cells; **b** and **d**, CD8^{+} T-cells) ten days after the final vaccination and immune responses were evaluated similar to post-challenge responses. Bars represent means and standard errors of the mean (SEM) (n = 7 to 12 mice in each vaccine group).

Supplementary Figure S8

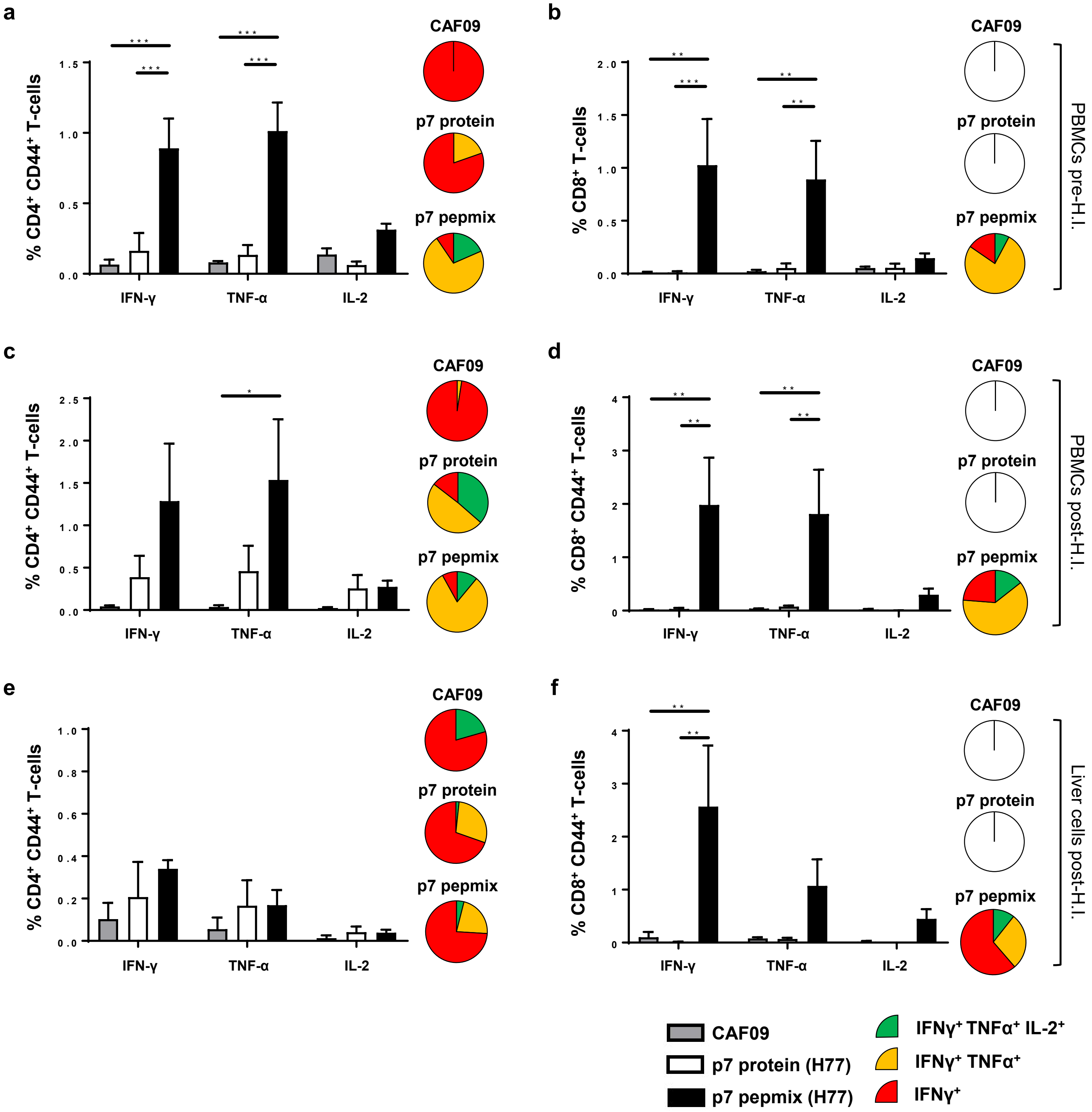

<u>Supplementary Figure S8</u>

Immune responses in vaccinated control mice "challenged" with PBS. Randomly selected mice from each vaccine group received PBS by hydrodynamic injection. The immune responses in these mice were assessed in parallel with those challenged with p7(H77)-GFP (Fig. 4 and 5). Frequencies of cytokine producing CD4$^+$ (**a**, **c** and **e**) and CD8$^+$ (**b**, **d** and **f**) are shown for T cell analyzed seven days before- (**a** and **b**) and five days after hydrodynamic injection (H.I.) with PBS (**c** and **d**). Similarly, cytokine responses of T-cells isolated from the liver were analyzed after hydrodynamic injection (**e** and **f**). Pie charts show the distribution of IFN-γ positive CD44$^+$ CD4$^+$ or CD44$^+$ CD8$^+$ T-cells divided into three subpopulations based on their ability to co-produce TNF-α alone or in combination with IL-2. Bars represent means and standard errors of the mean (SEM) (n = 3 to 4 mice in each vaccine group). *, $P < 0.05$; **$P < 0.01$; ***$P < 0.001$.

Supplementary Table S1.

In silico prediction of $CD8^+$ T cell epitopes within p7 peptide #4 in CB6F1 mice

HCV strain	Epitope sequence	Allele	MHC-I IC50 (nM)
J4	AAYAFYGVWPL	H-2-K^b	11.4
J4	YAFYGVWPL	H-2-D^b	42.8
H77	AVYAFYGM	H-2-K^b	5.6
H77	YAFYGMWPL	H-2-D^b	38.6

Supplementary Table S1. Prediction of strong $CD8^+$ T cell epitopes defined by the highest total score for proteasomal processing, TAP transport and MHC class I binding with IC50 values ≤ 500 nm were computed for H-2-K^b, H-2-D^b, H-2-K^d, H-2-D^d, H-2-L^d alleles.
